# Mitochondrial DNA of pre‐last glacial maximum red deer from NW Spain suggests a more complex phylogeographical history for the species

**DOI:** 10.1002/ece3.3553

**Published:** 2017-11-07

**Authors:** Alba Rey‐Iglesia, Aurora Grandal‐d'Anglade, Paula F. Campos, Anders Johannes Hansen

**Affiliations:** ^1^ Centre for Geogenetics Natural History Museum of Denmark University of Copenhagen Copenhagen Denmark; ^2^ Instituto Universitario de Xeoloxía “Isidro Parga Pondal” ESCI University of A Coruña A Coruña Spain; ^3^ CIMAR/CIIMAR Centro Interdisciplinar de Investigação Marinha e Ambiental Terminal de Cruzeiros do Porto de Leixões Universidade do Porto Matosinhos Portugal

**Keywords:** ancient DNA, Denmark, Late Pleistocene, phylogeography, red deer, Spain

## Abstract

The major climatic oscillations that characterized the Quaternary had a great influence on the evolution and distribution of several species. During cold periods, the distribution of temperate‐adapted species became fragmented with many surviving in southern refugia (Iberian, Italian, and Balkan Peninsulas). Red deer was one of the species that contracted its original range to southern refugia. Currently, two main lineages have been described for the species: western and eastern. We have analyzed fossils pre‐dating the last glacial maximum (LGM) from Liñares cave (NW Spain) that belongs to the peripheral range of the western clade, and fossils from the Danish Holocene belonging to the central part of the same clade. Phylogenetic analyses place our samples in the western clade. However, some specimens from Liñares represent an early split in the tree along with other pre‐LGM western samples from previous studies. Despite low bootstrap values in the Bayesian phylogenies, haplotype networks connect these foreign haplotypes to the eastern clade. We suggest a mixed phylogeographical model to explain this pattern with range expansions from the east during the expansion phase after the cold periods in marine isotope stage 3. We find slight isolation by distance in post‐LGM populations that could be a consequence of the recolonization from southern refugia after the LGM.

## 1 INTRODUCTION

Over the past ca. 2 million years, Earth has undergone drastic climatic oscillations (Provan & Bennett, 2008) influencing the evolution and distribution of several megafauna species (e.g., Campos et al., 2010; Hewitt, 1996; Hofreiter et al., 2004; Lister, 2004; Shapiro et al., 2004). During cold periods, climatic cooling and the associated spread of tundra ecosystems enabled the population and range expansion of arctic and subarctic species (e.g., Hewitt, 1996; Stewart & Lister, 2001). Meanwhile, temperate species distributions became fragmented and often limited to southern refugia like the Iberian, Italian, and Balkan Peninsulas in Europe. One of such temperate species is the red deer (*Cervus elaphus* L.). The species went through several environmental changes, likely involving genetic bottlenecks as a consequence of population reductions and/or fragmentation (Lister, 1984). Red deer fossils have been found in several habitats (Lister, 1984), suggesting that the species has a broad tolerance to different conditions (Sommer et al., 2008). This may have enabled red deer to benefit from the landscape changes brought about by glaciations, deglaciations, megafaunal extinctions, and, in recent times, human disturbance (Geist, 1998; Skog et al., 2009). In the past 10 years, several studies have dealt with the phylogeography of red deer in Europe (Ludt, Schroeder, Rottmann, & Kuehn, 2004; Meiri et al., 2013; Skog et al., 2009; Sommer et al., 2008; Zachos & Hartl, 2011). Based on mitochondrial DNA (mtDNA) from extant specimens, three main lineages can be defined: a western (lineage A), an eastern (lineage C), and a North African and Sardinian lineage (lineage B) (Skog et al., 2009; Zachos & Hartl, 2011). Western and eastern lineages show continuity at least until the Late Pleistocene (Meiri et al., 2013).

In an effort to increase the knowledge of the phylogeography and genetic diversity of Pleistocene red deer, we have sequenced mitochondrial genomes of pre‐LGM specimens from Spain. This region is in the peripheral limit of the western red deer clade and had a major role during the LGM as refugium. Red deer recolonized Europe after the LGM from this refugium (Meiri et al., 2013) and, as it has already been suggested by other authors, the genetic glacial composition of the species in this area presented relict foreign haplotypes (Meiri et al., 2013). At present, Spanish red deer populations can be grouped into two distinct maternal lineages (Sc and Sw) connected to two refugial areas in the Iberian Peninsula during the Würm glacial period (Carranza, Salinas, Andrés, & Pérez‐González, 2016; Fernández‐García, Carranza, Martínez, & Randi, 2014). It has been suggested that only one of these lineages (Sc) contributed to the northwestern European postglacial recolonization (Carranza et al., 2016).

In order to gain more understanding of the genetic composition pre‐dating the glacial period in Spain, we have studied specimens from Liñares cave (NW Spain). The fossil preservation in the cave is excellent (López‐González, Grandal‐d'Anglade, & Vidal‐Romaní, 2006), which has permitted the identification of the vast majority of fossil remains found in the cave. One of the most interesting features of the fossil composition is the presence of *Cervus elaphus*. A minimum number of 15 individuals have been calculated from the skull remains (López‐González, 2003). All the individuals have been identified as juvenile and senile males (López‐González, 2003). Red deer presence in karstic caves is well documented (López‐González, 2003); however, all these cases are caused by human action (López‐González, 2003). In Liñares, the accumulation of such a high number of deer specimens has been interpreted as a single episode of a group of animals searching for shelter (López‐González, 2003). Radiocarbon (^14^C) dating of these remains places them between 35 and >38,000 years before present (yBP) (López‐González, 2003; López‐González, Grandal‐d'Anglade, & Vidal‐Romaní, 1997).

In this study, we have analyzed red deer fossil remains from Liñares cave. We aimed to understand the genetic composition of the cave and place these individuals in the red deer European context. We also aimed to increase the knowledge in the Late Pleistocene red deer population structure and test the hypothesis of isolation by distance to explain spatial patterns. In order to complete the available data from different geographical regions (e.g., improve the understanding of spatial patterns), samples from the Danish Holocene were also included in the study, as no ancient samples from this period were available to date. Combining all these results, we suggest a mixed phylogeographical model to explain the species past haplotype distribution.

## 2 METHODS

### 2.1 Sampling site and specimens

**Figure 1:**
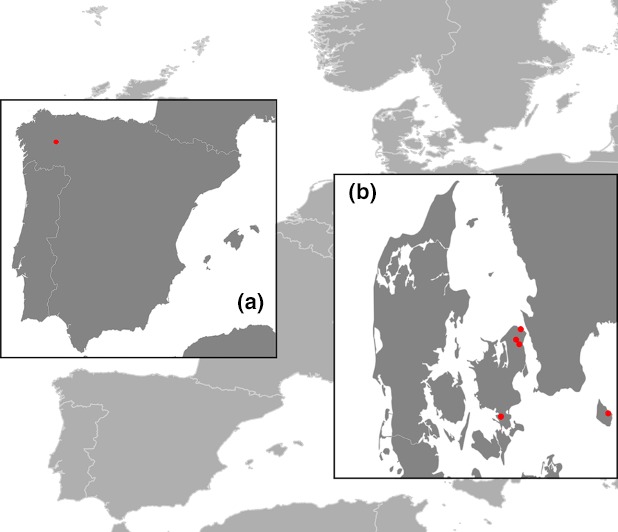
Map with the sampling sites. Liñares site is represented in (a). Sampling sites in Denmark are represented in inset (b)

In this study, we have included 30 ancient red deer fossils, 25 specimens collected from Liñares cave (NW Spain), and five individuals collected in Denmark (Figure 1). We sampled two regions within the western clade, a peripheral area (Liñares) and a more central region (Denmark). In addition, one modern specimen from Denmark was included in the study to generate the capture baits. Permissions were obtained from all museums and institutions to access the collections and all samples were on loan for scientific purposes. Detailed information from all the specimens included in the study can be found in Table S1.

### 2.2 Extractions ancient samples

All the pre‐amplification steps were performed in a specialized ancient DNA facility that is physically isolated from the location where post‐PCR‐amplified products are manipulated. To guard against contamination, all reagents used were of molecular biology grade, and blank extraction controls were used.

An average of 150 mg of bone powder was drilled from the different ancient specimens. Two different extraction methods were used (Table S1).

DNA from some of the samples was extracted from bone powder using a silica column‐based method (Yang, Eng, Waye, Dudar, & Saunders, 1998). Bone powder was mixed with 1.3 ml 0.5 M EDTA pH 8 (Invitrogen) and 2 mg/ml of proteinase K (Invitrogen) and incubated on a shaker at 37°C overnight. After overnight lysis, samples were centrifuged at maximum speed for 5 min in order to pellet the nondigested powder. The supernatant was then transferred to a 15‐ml 30‐kDa Amicon centrifugal filter unit (Millipore) and spun at 4,000 × *g* for 10 min or until the liquid was concentrated down to about 200–250 μl. DNA was then purified with MinElute columns (Qiagen), following the manufacturer's instructions with the exception of a 15‐min incubation at 37°C during the elution step. For other samples, DNA was extracted following the silica particle‐based protocol. A total of 150 mg bone powder was digested for 24 hours at 37°C in a 5‐ to 10‐ml buffer consisting of 0.5 M EDTA (Invitrogen), 0.5% N‐lauryl sarcosyl (Merck), and 2 mg/ml proteinase K (Invitrogen). The digestion buffer containing released DNA molecules were recovered following centrifugation at 500g for 5 min and further purified using a silica particle‐based approach as described in Orlando et al. (2009).

### 2.3 Extractions modern sample

DNA was extracted from fresh muscle using the DNeasy Blood and Tissue kit (Qiagen) following the manufacturer's instructions. Samples were left to lyse overnight at 55°C. Prior to elution, samples were incubated at 37°C for 15 minutes. Elution volume was 100 μl.

### 2.4 Quantitative PCR

A quantitative PCR (qPCR) assay was used to screen for red deer on the DNA extracts (Bunce, Oskam, & Allentoft, 2012) using a primer pair spanning 133 bp of the mitochondrial control region (CR4; Meiri et al., 2013; Table S2). Quantitative PCR amplifications consisted of 1.5U HiFi Platinum Taq (Invitrogen, Life Technologies), 1× HiFi buffer, 2 mM MgSO4, 0.25 mM dNTPs, 0.2 μM of each primer, SYBR Green dye 0.6 μl, and 2 μl of DNA. PCR cycle conditions consisted of an initial denaturation for 4 minutes (min) at 94°C, followed by 55 cycles of 60‐second (s) denaturation at 94°C, 60‐s annealing at 46°C, and 60‐s elongation at 68°C, including a final elongation for 5 min at 68°C. Each sample was replicated three times.

### 2.5 Libraries

DNA libraries were built using the NEBNext DNA Library Prep Master Mix Set for 454 (New England BioLabs, ref: E6070). A volume of 21.5 μl of DNA extract was used in the end‐repair step. Blunt‐end adaptors at 25 μM concentration (Meyer & Kircher, 2010) were added in the ligation reactions. Adapter fill‐in reaction was performed for 20 min at 65°C and 20 min at 80°C to inactivate the enzyme. All the steps were performed in 25 μl volume reactions. DNA was purified after end‐repair and blunt‐end steps with MinElute (Qiagen). Library‐indexing PCR was performed on each library in 50 μl. The PCR consisted of 5U HiFi Platinum Taq (Invitrogen, Life Technologies), 1× HiFi buffer, 1.5 mM MgSO4, 0.2 mM dNTPs, 5 μM of Primer lnPE1.0, 5 μM of an index primer containing a unique 6‐nucleotide index tag, and 15 μl of library. PCR cycle conditions consisted of an initial denaturation for 60 s at 94°C, followed by 13–16 cycles of 30‐s denaturation at 94°C, 30‐s annealing at 55°C, and 30‐s elongation at 68°C, including a final elongation for 5 min at 68°C. Amplified libraries were concentrated using a MiniElute column. Subsequently, DNA concentration was measured using both the Qubit Fluorometer (HS) and the Agilent 2100 Bioanalyzer (BA). Library re‐amplifications were performed for some of the libraries to reach the 1,000 ng of starting material required for capture as in Maricic, Whitten, and Pääbo (2010). Re‐amplifications were conducted in two reactions per sample using 7.5 μl of indexed library on each reaction and eight cycles of PCR. Both reactions were pooled and cleaned up together using a MiniElute column. Subsequently, DNA concentration was measured using both the Qubit Fluorometer (HS) and the Agilent 2100 Bioanalyzer.

### 2.6 Mitochondrial capture

Mitochondrial captures were performed as in Maricic et al. (2010). First, mitogenomes from a modern red deer sample were generated using four primer pairs spanning the entire length of the mitochondrion (Wada, Okumura, Nishibori, Kikkawa, & Yokohama, 2010; Table S2). The PCR consisted of 5U HiFi Platinum Taq (Invitrogen, Life Technologies), 1× HiFi buffer, 1.5 mM MgSO4, 0.25 mM dNTPs, 0.4 μM of each of the primers, and 2–5 μl of DNA depending on the quality of the extract. PCR cycle conditions consisted of an initial denaturation for 2 min at 94°C, followed by 40 cycles of 30‐s denaturation at 94°C, 30‐s annealing at 52°C, and 5‐min elongation at 68°C, including a final elongation for 7 min at 68°C. A gel was run for the different reactions to check for successful amplification. Following PCR amplification, amplicons were purified and quantified using a Qubit, and then pooled at equimolar concentrations. Subsequently, the DNA was sheared and biotinylated adaptors were added. Indexed libraries were denatured and incubated with the biotinylated bait. Subsequent streptavidin capture allowed us to target for mitochondrial sequences. For full details on the method, see Maricic et al. (2010). Amplified libraries were quantified using the 2100 BA High‐Sensitivity DNA Assay. Libraries were multiplexed and paired‐end sequenced on Illumina MiSeq platform at the Danish National High‐Throughput DNA Sequencing Centre.

### 2.7 Sequence processing

The dataset consisted of 27,073,487 paired‐end reads. Raw reads were processed using AdapterRemoval (Lindgreen, 2012), trimming low‐quality bases, and discarding reads shorter than 30 bp. Only collapsed reads with an overlap of 11 bp were used for downstream analysis. Reads were mapped against a red deer mitochondrial genome (AB245427.2; Wada et al., 2010) using BWA (Li & Durbin, 2009) with default options except for disabled seed. Reads mapping to the reference were sorted and duplicates removed using Samtools (Li et al., 2009). Alignments were visually inspected using Geneious v7 (http://www.geneious.com; Kearse et al., 2012) and a consensus sequence was called using the “strict” consensus option for each of the BAM files. Table S1 contains information about reads, coverage, and depth for each sample. MapDamage2.0 (Jónsson, Ginolhac, Schubert, Johnson, & Orlando, 2013) was used to analyze the damage pattern in the fragments. See Figure S1 for damage pattern misincorporation.

### 2.8 Phylogenetic analyses

Phylogenetic relationships were estimated using maximum likelihood (ML), Bayesian phylogenies, and median‐joining haplotype networks. One of the limitations when working with nonmodel species is the availability of datasets for comparison. In this way, two phylogenies were created. A mitogenome phylogeny of the *Cervus* genus, including one representative of the different *Cervus* species available in the NCBI (Table S3) and a combined control region (CR) and cytochrome b (Cytb) red deer phylogeny. For the *Cervus* genus phylogeny, an alignment of 17,047 bp was built in Geneious v7 using MAFFT algorithm (Katoh, Misawa, Kuma, & Miyata, 2002). A maximum‐likelihood phylogeny was created using PhyML online (http://atgc.lirmm.fr/phyml/). The best substitution model was selected in jModelTest 2 (Darriba, Taboada, Doallo, & Posada, 2012) under the Bayesian information criterion (HKY+I+G, ti/tv 6.8562, p‐inv 0.41, and shape 0.6460).

For the red deer phylogeny, our data was combined with the dataset generated by Meiri et al. (2013). So far, this is the most comprehensive dataset for red deer, including modern and ancient samples. The dataset contains a concatenate of 784 bp of the CR and Cytb. Our mitogenomes were loaded in Geneious v7 and trimmed to be comparable with Meiri et al. (2013) haplotypes. See Table S4. For ML, the best nucleotide substitution model was selected using the Bayesian information criterion in jModelTest 2 (Darriba et al., 2012). The best‐fitting model for our sequences was HKY+I+G with values of 16.5315 for transition/transversion ratio, 0.5240 for the portion of invariable sites, and 0.7640 for the gamma distribution. ML phylogeny was created using PhyML online (http://atgc.lirmm.fr/phyml/; Guindon et al., 2010). Markov chain Monte Carlo (McMC) sampling was performed in MrBayes v.3.2 (Ronquist & Huelsenbeck, 2003). The concatenate alignment was partitioned in CR, Cytb first and second codon, and Cytb third codon, as determined using PartitionFinder (Lanfear, Calcott, Ho, & Guindon, 2012), and the best‐fitting model was selected for each partition using jModelTest 2 (Darriba et al., 2012). McMC sampling was performed in two separate analyses with four chains of 10 million generations, sampling every 1,000 generations, and discarding the first 25% as burn‐in. Haplotype networks for the 784‐bp alignment were constructed using a median‐joining algorithm (Bandelt, Forster, & Röhl, 1999) as implemented in Network 4.6 (http://www.fluxus-engineering.com/sharenet.htm). Haplotype files were preprocessed using the star contraction option. Median‐joining networks were postprocessed using the MP option (Polzin & Daneshmand, 2003) to eliminate unnecessary median vectors and links.

### 2.9 Haplotype chronology

In order to review the haplotype chronology and temporal genetic continuity of the species, a database for all the published sequences for red deer mtDNA was created. A final alignment of 567 sequences from the CR (positions 15,573–15,751; reference: AB245427) with a length of 179 bp was created. Haplotypes for those sequences were obtained in DnaSP v5 (Librado & Rozas, 2009). Haplotypes were imported into TempNet (Prost & Anderson, 2011) and divided into three temporal windows: pre‐LGM (>16 kyr), post‐LGM (<16 kyr – 500 years), and modern samples. Only those haplotypes that represented more than one sequence were included in the TempNet analysis to improve the visualization. See Table S4.

### 2.10 Isolation by distance

Geographical structure in past red deer populations was investigated by correlating genetic distances to geographical distances. Our data were combined with data from Meiri et al. (2013); see Table S4 for list of specimens included in this analysis with dates and geographical position. Pairwise nucleotide distance matrices were produced in R with the package ape (Paradis, Claude, & Strimmer, 2004). Matrices were calculated for nine different datasets: concatenated CR and Cytb for all the ancient samples (pre‐ and post‐LGM), and pre‐LGM and post‐LGM samples. Matrices were also obtained for the CR and Cytb fragments individually. Geographical distance matrices were calculated in kilometers using the Geographical Distance Matrix Generator (Ersts, 2011) and, then, log‐transformed in R. Isolation by distance was tested using Mantel test in R with the package adegenet (Jombart, 2008) for the different datasets.

### 2.11 Iberian Peninsula

In order to investigate the change in genetic diversity for Spanish red deer populations in the last ca. 40,000 years, DnaSP v5 (Librado & Rozas, 2009) was used to calculate nucleotide diversity (π), haplotype diversity (Hd), number of polymorphic sites (S), and Tajima's D (Table 2). Once again, in this analysis, our Spanish samples were combined with those from Meiri et al. (2013) (See Table S4).

## 3 RESULTS

### 3.1 Extractions, quantitative PCR, mitogenomes

We extracted DNA from 25 red deer samples from Liñares cave and five from Denmark. Extracts were screened with a red deer mitochondrial qPCR assay. Samples that presented a consistent amplification were built into Illumina libraries, enriched for mitochondrial sequences, and then sequenced in a MiSeq platform. We have generated 12 complete mitogenomes (defined as >90% of the genome, covered to at least 3× unique read depth) and four partial mitogenomes. GenBank accession numbers for these sequences are MF872236‐MF872249, see Table S1.

### 3.2 Phylogenies

**Figure 2:**
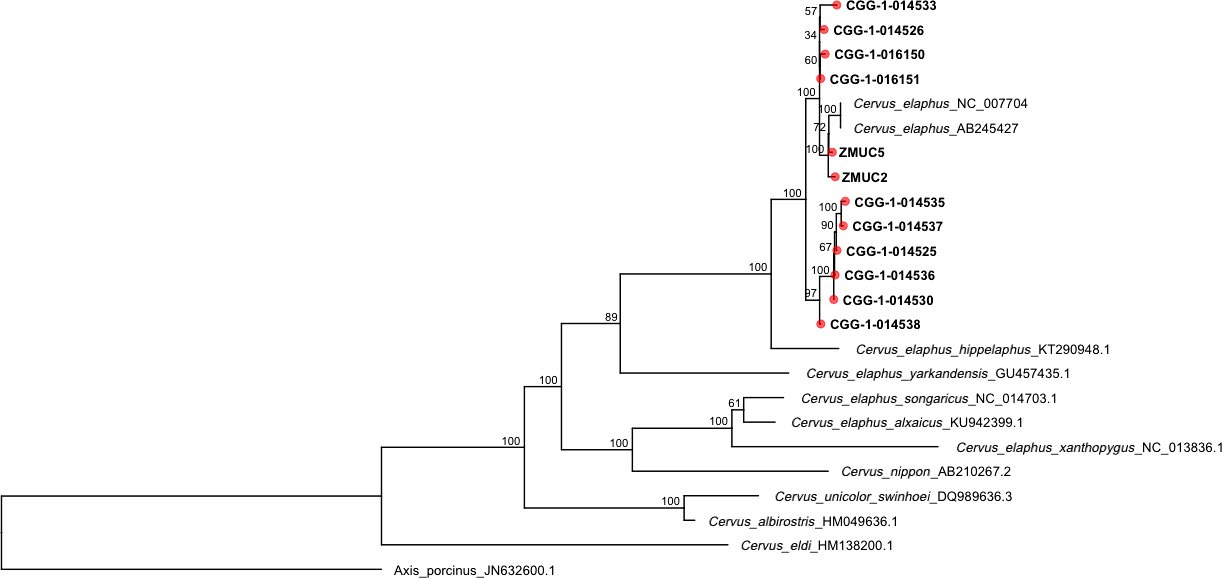
Maximum‐likelihood phylogeny for the *Cervus* genus. The phylogeny represents a mitochondrial alignment of 17,047 bp. The specimens from this study are indicated in bold and with a red circle in the tip. Our samples lie within the red deer clade (*C. elaphus*) representing two well‐supported clades. *Axis porcinus* was used as an outgroup

In the 17,047‐bp *Cervus* mitogenome phylogeny, our samples are grouped within the red deer clade (Figure 2). The specimens are divided into two well‐supported clades, indicating two potential lineages within our samples. The first clade includes four pre‐LGM individuals from Liñares (CGG‐1‐014526, CGG‐1‐014533, CGG‐1‐016150, and CGG‐1‐016151), two Holocene samples from Denmark, and two western red deer references. The second clade groups the remaining six haplotypes present at Liñares (CGG‐1‐014525, CGG‐1‐01430, CGG‐1‐014535, CGG‐1‐014536, CGG‐1‐014537, and CGG‐1‐014538). Within the clades, there is low genetic structure, except for the Danish samples being more closely related to the modern references included in the tree.

**Figure 3:**
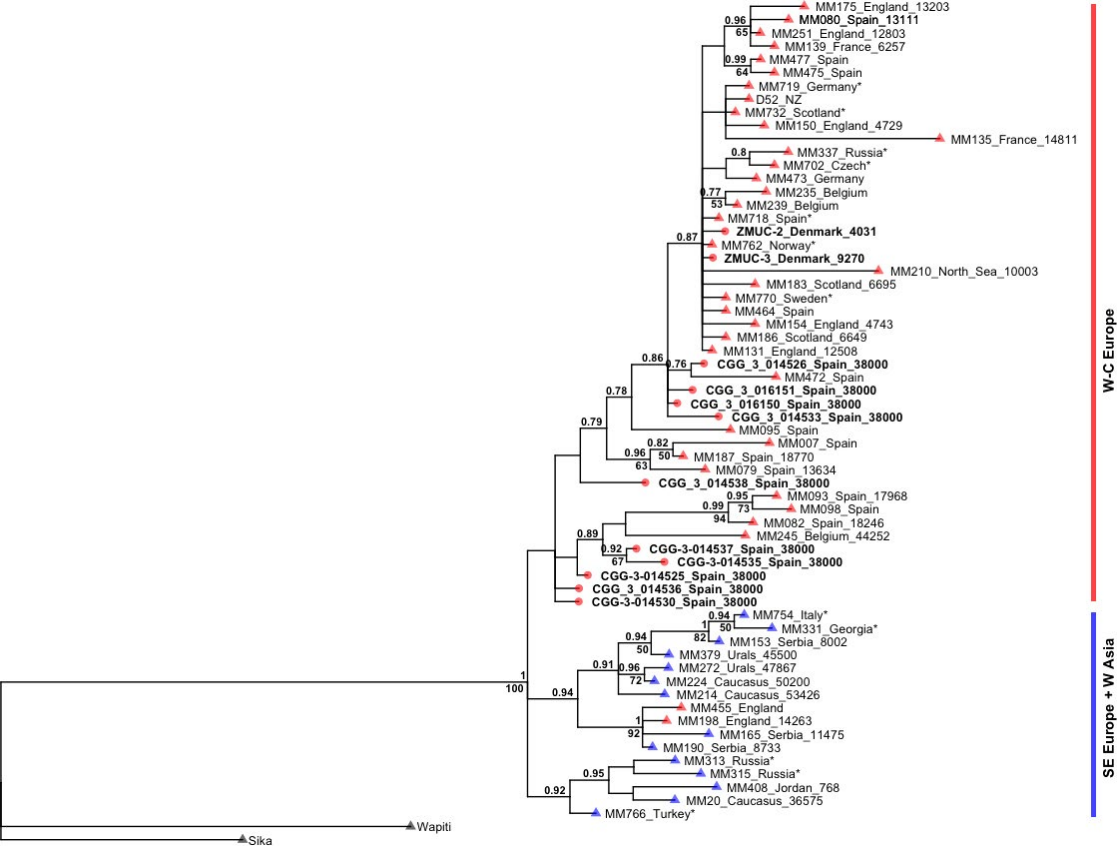
Phylogenetic tree obtained in MrBayes based on 784 bp of concatenated Cytb and CR sequences. Tip point colors correspond to the geographical location of the samples. Red: Western/Central Europe. Blue: southeast Europe and Western Asia. Samples from this study are indicated in bold and with a circle in the tip point. Sika deer (MM714) and Wapiti (MM722) deer were used as outgroups. Samples with * indicate modern specimens. Radiocarbon dates were added to the tip of the tree when available for the ancient specimens. Bootstrap (>50) and posterior probabilities (>0.75) are shown in the branches

**Figure 4:**
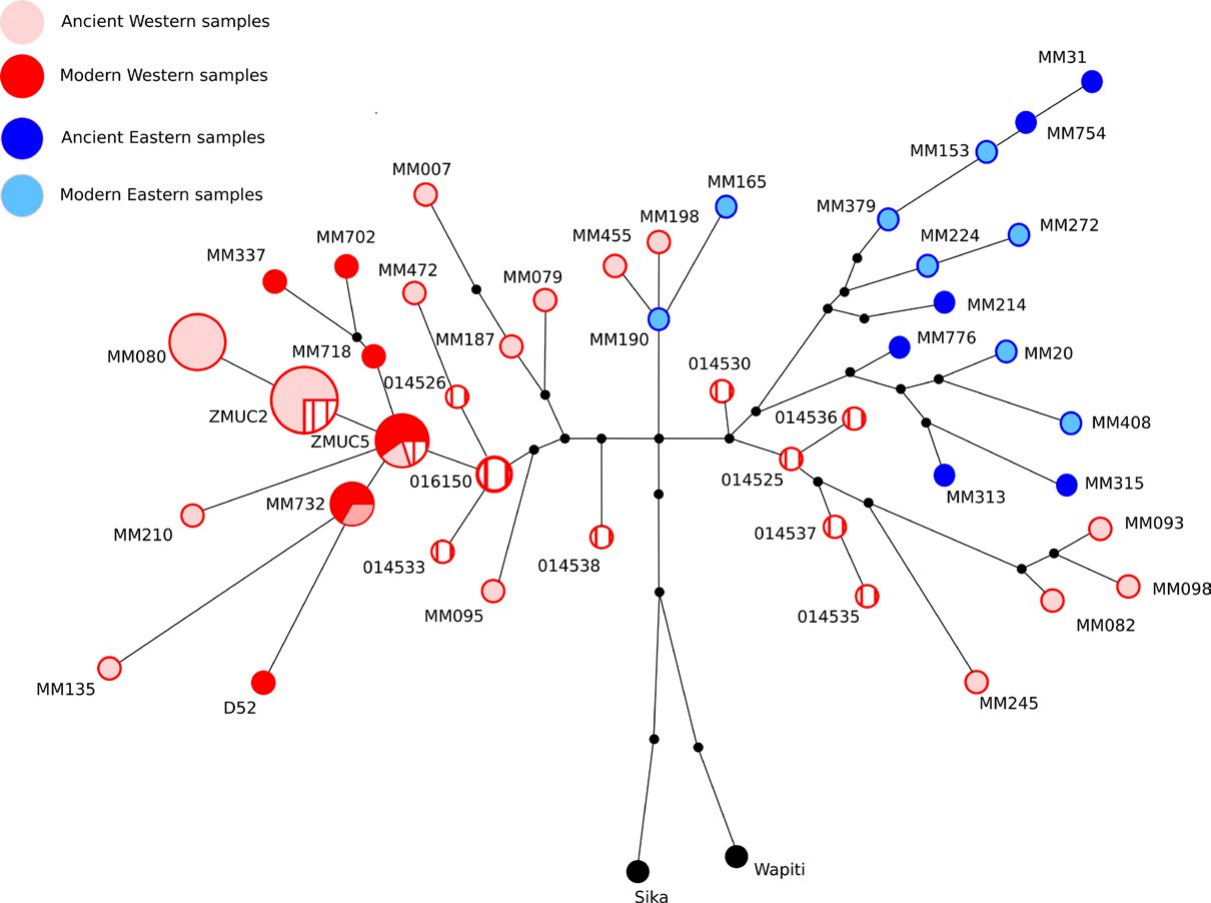
Rooted haplotype network built using a MJ algorithm under Network 4.6 representing 65 sequences and 48 different haplotypes of 784 bp. The circles with larger size represent haplotypes that were merged by the star contraction algorithm (MM080, ZMUC2, ZMUC5, MM732, and CGG‐1‐016150). The colors correspond to geographical location of the samples. Red: Western/Central Europe. Blue: southeast Europe and Western Asia. The samples from this study are represented by a cross pattern. Sika deer and wapiti were used as outgroup for rooting the network

The ML and Bayesian analyses for the 784‐bp CR and Cytb concatenate present similar topologies (Figure 3). Our samples cluster in the Western and Central European groups as defined by Meiri et al. (2013). In their study, two early splits in the tree are described. These splits were represented by a small group of three Spanish samples (MM082, MM093, and MM098; from 17,968 to 18,662 cal. yr BP), and one old Belgian sample (MM245; 44,252 cal. yr BP). Five of our samples (CGG‐1‐014525, CGG‐1‐01430, CGG‐1‐014535, CGG‐1‐014536, and CGG‐1‐014537) form a group with these four haplotypes. The support for this group is, however, very low (0.64 pp). These five samples also cluster together in a separate clade in the *Cervus* phylogeny (Figure 2). The haplotype network for Cytb and CR shows two haplotype clusters (Figure 4): a Western/Central European and a Eastern European and Asian cluster. In the network, five of our haplotypes lie within the Eastern/Asian cluster: CGG‐1‐014525, CGG‐1‐014530, CGG‐1‐014535, CGG‐1‐014536, and CGG‐1‐014537.

The low bootstrap in the 784‐bp CR and Cytb concatenate phylogeny (Figure 3) is probably due to the length of the alignment used to build this phylogeny. The reduced number of single nucleotide polymorphisms (SNPs) between the haplotypes would decrease the resolution of the tree. The high clade support in the *Cervus* mitogenome phylogeny (Figure 2) serves as an example of this. Using whole mitogenomes, the relationship between the mitochondrial sequences generated in this study can be better assessed, as there is an increase in the number of SNPs. Finally, haplotype networks are powerful methods for estimating intraspecific relationships as they can incorporate and display low divergence, extant ancestral nodes, multifurcations, and reticulations (Posada & Crandall, 2001).

### 3.3 Haplotype chronology

**Figure 5:**
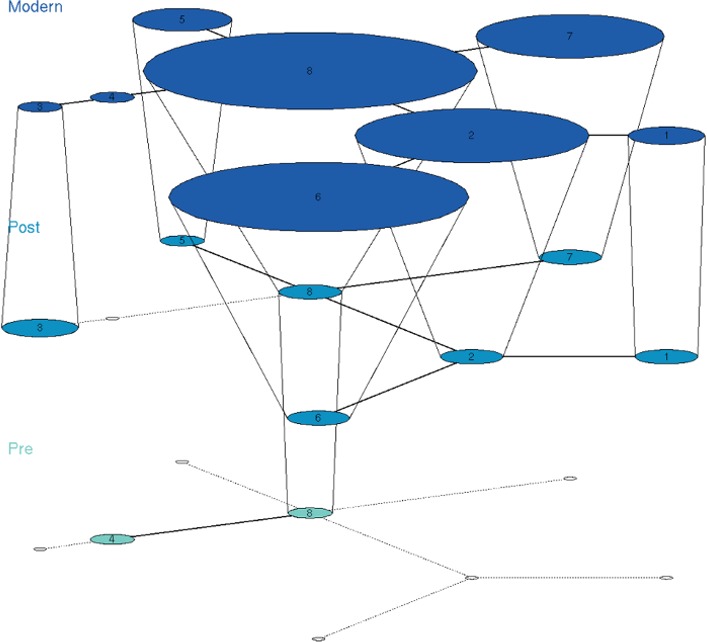
Haplotype network obtained in TempNet for 179 bp of the CR. This network represents all the haplotypes obtained in DnaSP v.5 that contain more than one sequence. To improve the visualization, we have only included those haplotypes that are shared across temporal layers. The temporal layers for the analysis are pre‐LGM (>16 kyr, light blue), post‐LGM (<16 kyr, blue), and modern (dark blue)

The final dataset for this analysis included 567 sequences of 179 bp for the CR. These sequences represent 154 haplotypes, 94 of them represented by single sequences. There are 61 ancient red deer haplotypes that are not present in extant populations, with the period >16 kyr presenting the largest haplotype and nucleotide diversity. The temporal network from Figure 5 shows that haplotype continuity is very limited, and few haplotypes are connected across layers. Two individuals from Liñares present haplotypes that are still present in modern red deer. Specimen CGG_1_016150 (represented as haplotype 6) harbors one of the most common haplotypes in extant western red deer (Table S4), and this haplotype is also found in Holocene samples from Norway and one of our Holocene Danish samples (ZMUC_5). The other pre‐LGM specimen showing lineage continuity is CGG_1_014526 (represented as haplotype 4), and the same haplotype is present in two modern red deer from Poland (Table S4). The Danish haplotype ZMUC2 is also present in the modern genetic pool, in a cluster that combines modern Danish and Norwegian samples. Other post‐LGM sequences published in previous studies also show lineage continuity with modern haplotypes (Table S4).

### 3.4 IBD

We detected statistically significant isolation by distance for the datasets all ancient and post‐LGM. The full pre‐LGM dataset was not statistically significant for IBD. (Table 1). Regression coefficients were very low for all the models (see Table 1).

**Table 1:** Results from the Mantel test for the correlation of genetic and geographical matrices

	All ancient samples			Pre‐LGM and LGM 38–16 kyr			Post‐LGM (16 kyr – Holocene)		
	Full	CR	Cytb	Full	CR	Cytb	Full	CR	Cytb
*p*‐value	.006*	.005*	.114	.418	.291	.615	.011*	.026*	.026*
*R* ^2^	0.0831	0.0953	0.0323	0.0005	0.0080	0.0031	0.1405	0.1214	0.09865

Significant values are marked with *.

### 3.5 Genetic diversity in the Iberian Peninsula

The 28 Spanish sequences for the combined CR and Cytb (784 bp) dataset yield 23 haplotypes (Table 2). Haplotype diversity is (Hd) 0.981 and nucleotide diversity (π) is 0.00967. Neutrality tests are negative (Tajima′s D neutrality: −1.1835) and nonsignificant, indicating neutral evolution.

**Table 2:** Genetic diversity in the Iberian Peninsula. Tajima's D values are nonsignificant

Dataset	bp	*N*	*h*	Hd	Hd variance	Std	π	Tajima's D
All	784	28	23	0.981	0.00025	0.016	0.00969	−1.1835
>16,000 cal. Yr BP	784	16	16	1	0.00049	0.022	0.00977	−1.03299
<16,000 cal. Yr BP	784	12	7	0.879	0.00565	0.075	0.00702	−0.71929

## 4 DISCUSSION

In this study, we have generated new pre‐LGM and Holocene data for red deer in Spain and Denmark, respectively. The Spanish pre‐LGM data suggest that the phylogeographical model for the species before the LGM was more complex than previously assumed and might not be only explained by lack of phylogeographical structure (Meiri et al., 2013; Niedziałkowska et al., 2011) as it has been hypothesized. All the Spanish red deer samples were collected at the karstic cave of Liñares with radiocarbon dates prior to the LGM (ca. 38,000 years). This is consistent with other previous studies where radiocarbon dates show that red deer was already present in Europe pre‐dating the LGM (Meiri et al., 2013). The specimens from the cave present a rather diverse haplotype configuration. Half of the haplotypes from Liñares fit in the Western European clade, which is in line with previous red deer studies (Ludt et al., 2004; Meiri et al., 2013; Skog et al., 2009; Sommer et al., 2008; Zachos & Hartl, 2011) and also with data from other species (Hofreiter et al., 2004; Fortes et al., 2016; Niedziałkowska, 2017). For instance, studies on cave bear suggest some gene flow between the Iberian Peninsula, France, and Central Europe during marine isotope stage 3 (MIS 3, 59,000–24,000 BP) (Fortes et al., 2016). The rest of the specimens from Liñares are connected to a pre‐LGM Belgium sample from Trou Al'Wesse Cave dated as 44,252 years old) and to three LGM Spanish haplotypes (Figures 2 and 3). This Spanish–Belgium group has been shown to be connected with the southeast European and Western Asian clade (Meiri et al., 2013; also known as lineage C). Similar patterns have been reported for other species (García‐Vázquez, Pinto Llona, & Grandal‐d'Anglade, 2017; Massilani et al., 2016; Valdiosera et al., 2008). García‐Vázquez et al. (2017) report the presence of brown bear haplotypes from clade 3c and clade 4 in two samples dated to ca. 31,000 and ca. 30,000, respectively, in the NW part of Spain. Brown bear clade 3c and clade 4 had only been previously described in North America (clades 3c and 4) and Japan (clade 4; García‐Vázquez et al., 2017).

Two different scenarios might explain the presence of the eastern red deer haplotypes in Western Europe. One scenario would be a less prominent structure of red deer populations in Europe before the LGM as suggested by Hofreiter et al. (2004) and Meiri et al. (2013). The lack of phylogeographical structure has been previously accepted for other species, for example, hyenas, bears, and Neanderthals (Hofreiter et al., 2004; Valdiosera et al., 2008), red fox (Teacher, Thomas, & Barnes, 2011), and reindeer (Lorenzen et al., 2011). This model suggests that prior to the LGM, the phylogeographical patterns in European mammals were very reduced, with extensive mixing between populations in most of the range outside the refugia during interglacial periods (Hofreiter et al., 2004). The lack of structure would explain the presence of foreign haplotypes in Western Europe. Based on our analysis, the modern and post‐LGM datasets reject the null hypothesis of random arrangement (i.e., existence of population structure derived from the refugia in the recolonization phase). However, the lack of phylogeographical structure could not be rejected for the pre‐LGM dataset.

The other possible scenario that could explain the presence of foreign haplotypes in Western Europe would be that individuals carrying these haplotypes could have entered the Iberian Peninsula before the LGM, in the first stages of the glacial period as described for other faunas (Álvarez‐Lao & García, 2011; Grandal‐d'Anglade, López‐González, & Vidal‐Romaní, 1997) and extended toward the NW side of the Iberian Peninsula. This hypothesis would be in line with the new phylogeographical model suggested for brown bear where migration movements originated from a central Asian nucleus (García‐Vázquez et al., 2017). The pre‐LGM period was characterized by recurrent climatic changes including several episodes of advances and retreats of the polar ice caps and continental glaciers, with the Saalian glaciation (MIS 6) having had a greater extension than the LGM (Ehlers & Gibbard, 2007). The advance of ice during cold periods would constraint the populations while in the expansion phases the favorable condition would allow movements toward the west.

Our clade continuity analysis shows that sequences were merged in haplotypes that maintained the clade structure (i.e., western and eastern clades have been maintained in time with no big disturbances), suggesting that already in the Late Pleistocene there was a certain degree of structure present. This analysis also shows that two pre‐LGM individuals from Liñares present haplotype continuity in the modern red deer genetic pool (Figure 4). This confirms the role of northern Spain as a refugium and in the recolonization phase after the LGM, as already shown by Meiri et al. (2013). In their study, samples from El Miron and Mazaculos Cave (both in Northern Spain) form a clade with extant western red deer. None of the foreign haplotypes from Spain are shared with other temporal layers, which suggests that these haplotypes were probably present in very low frequency. The genetic diversity analyses show a decrease in haplotype and nucleotide diversity from the pre‐LGM to the post‐LGM period in Spain. This loss of diversity reflects the effect of the last glacial period in the red deer populations, with some lineages being lost throughout the LGM.

Our study site, Liñares cave, preserved a diverse aggregate of pre‐LGM red deer fossils. By studying this single site, we were able to confirm the role of Iberia as refugium during the LGM. Some of the red deer from this site also carried foreign haplotypes associated with eastern red deer diversity. It is the first time that these haplotypes have been described in Spain pre‐dating the LGM. It is important to mention the low statistical support for some of the clades in one of our phylogenies (Figure 3). However, when combining all the phylogenetic results, there is a robust evidence for suggesting the association of this foreign haplotypes with the eastern diversity. As we have hypothesized, the foreign haplotype presence might be explained by red deer movements from Eastern Europe during MIS‐3. Nevertheless, we do not have enough genetic data to confirm this model. Our data also indicate a decrease in genetic diversity in the Iberian Peninsula through time. Our results highlight the importance of pre‐LGM data to complete the phylogeographical model for species. Using high‐throughput sequencing combined with capture methods will allow us to obtain whole mitogenomes from more pre‐LGM specimens and, possibly, nuclear information that could be used to test the different phylogeographical scenarios suggested in this study.

## AUTHORS' CONTRIBUTION

A.R.I. carried out the molecular laboratory work, data analysis, and study design and drafted the manuscript; A.G.A. excavated Liñares cave, performed fossil identification, participated in the design of the study, and participated in manuscript drafting; P.F.C. participated in data analysis study and participated in manuscript modifications; A.J.H. participated in the design of the study and in manuscript modifications.

## CONFLICT OF INTEREST

None declared.
