# Supplementary material for: Mitochondrial DNA of pre‐last glacial maximum red deer from NW Spain suggests a more complex phylogeographical history for the species
Source: Ecol Evol. 2017 Nov 7;7(24):10690–700. doi: 10.1002/ece3.3553 (PMC5743481; doi:10.1002/ece3.3553)
Supplement: Supplementary file 1 [file ECE3-7-10690-s001.docx]

**
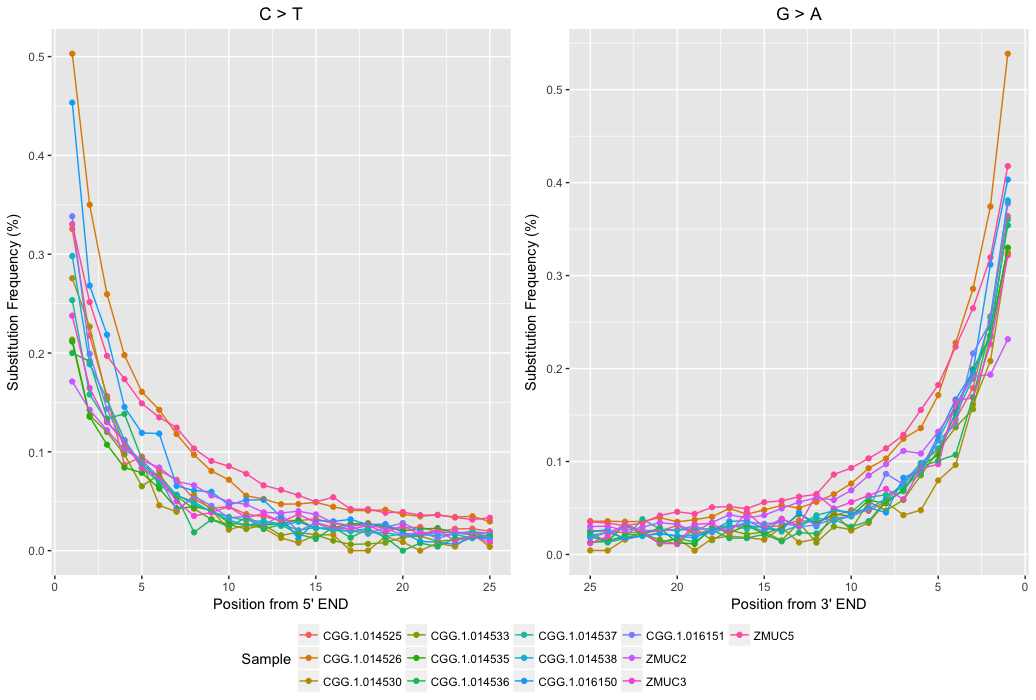
Figure S1** Misincorporation plot generated by mapDamage2 (Jónsson *et al*., 2013) for the complete mitogenomes included in this study. The plots show the substitution pattern at the 5’ and 3’ ends of the aligned reads to the reference mitochondrial sequence (AB245427.2; Wada *et al*., 2010).
