# Supplementary material for: Mitochondrial DNA of pre‐last glacial maximum red deer from NW Spain suggests a more complex phylogeographical history for the species
Source: Ecol Evol. 2017 Nov 7;7(24):10690–700. doi: 10.1002/ece3.3553 (PMC5743481; doi:10.1002/ece3.3553)
Supplement: Supplementary file 3 [file ECE3-7-10690-s003.docx]

| **Primer set** | **Forward (position)** | **Reverse (position)** | **Size** | **Reference** |
| --- | --- | --- | --- | --- |
| I | TAGCAATTATTCTACTATCCGTCCTC (3177–3206) | GAATTAGTAGGTGTCCTGCAGTAATGTTAG (8424–8453) | 5281 | 1 |
| II | TTATAGGCCTTCCACTAGCTACTCTC (7976–8005) | GTGATTGTGACTAGGAAAGAGAGGAAATAC (13 514–13 543) | 5570 | 1 |
| III | CTCTAATATACCCCTAATAGGCCTTG (12 378–12 407) | CTGAAGATGGCGGTATATAGACTGTATTAG (663–692) | 4674 | 1 |
| IV | AAGTTAATAAGACTAAGAGGAGCTG (148–176) | GTGGATAGAACAACTATTGTAGGTAGAAGG (4881–4910) | 4765 | 1 |
| V | TTATAGGCCTTCCACTAGCTACTCTC (7976–8005) | GAATTAGTAGGTGTCCTGCAGTAATGTTAG (8424–8453) | 477 | 1 |
| VI | TAGTACATTATATTATATGCCCCATG | GACGGGATACGCATGTTG | 133 | 2 |

**Table S2** Primer pairs used for qPCR testing of our extractions and generation of the mitochondrial baits for capture.

**References**

1. Wada, K., Okumura, K., Nishibori, M., Kikkawa, Y. & Yokohama, M. 2010. The complete mitochondrial genome of the domestic red deer (Cervus elaphus) of New Zealand and its phylogenic position within the family Cervidae. *Anim. Sci. J.* **81**: 551-557.

2. Meiri, M., Lister, A.M., Higham, T.F.G., Stewart, J.R., Strays, L.G., Obermaier, H. *et al.* 2013. Late‐glacial recolonization and phylogeography of European red deer (Cervus elaphus L.). *Mol. Ecol*. **22**: 4711-4722.
