# Supplementary material for: Mitochondrial DNA of pre‐last glacial maximum red deer from NW Spain suggests a more complex phylogeographical history for the species
Source: Ecol Evol. 2017 Nov 7;7(24):10690–700. doi: 10.1002/ece3.3553 (PMC5743481; doi:10.1002/ece3.3553)
Supplement: Supplementary file 4 [file ECE3-7-10690-s004.docx]

Table S3 - List of publicly available mitochondrial genome sequences (GenBank accession numbers and references) included in the *Cervus* mitogenome phylogeny (Figure 2).

| Species name | Accession number | Reference |
| --- | --- | --- |
| *Cervus elaphus* | NC_007704 | Olivieri *et al*., 2014 |
| *Cervus elaphus* | AB245427 | Wada *et al*., 2010 |
| *Cervus e. hippelaphus* | KT290948 | Frank *et al.,* 2016 |
| *Cervus e. yarkandensis* | GU457435 | Zha *et al.*, 2010* |
| *Cervus e. alxaicus* | KU942399 | Shi *et al.*, 2016* |
| *Cervus e. songaricus* | NC_014703 | Yu *et al.,* 2010* |
| *Cervus e. xanthopygus* | NC_013836 | Zha *et al.*, 2010* |
| *Cervus nippon* | AB210267 | Wada & Yokohama, 2007 |
| *Cervus albirostris* | HM049636 | Wu *et al.,* 2010* |
| *Cervus unicolor swinhoei* | DQ989636 | Chen *et al.,* 2011 |
| *Cervus eldi* | HM138200 | Kong & Li, 2010* |
| *Axis porcinus* | JN632600 | Hassanin *et al*., 2012 |

*unpublished
