# Supplementary material for: Mitochondrial DNA of pre‐last glacial maximum red deer from NW Spain suggests a more complex phylogeographical history for the species
Source: Ecol Evol. 2017 Nov 7;7(24):10690–700. doi: 10.1002/ece3.3553 (PMC5743481; doi:10.1002/ece3.3553)
Supplement: Supplementary file 5 [file ECE3-7-10690-s005.docx]

**Table S4 Information on sequences from other studies included in our analyses**

| **Analysis** | **Code** |
| --- | --- |
| Phylogeny | 1 |
| NJ Network | 2 |
| IsolationByDistance | 3 |
| Haplotype continuity | 4 |
| Diversity Spain | 5 |

|  |
| --- |

| **Original hap name** | **Hap (this study)** | **Ascension** | **Location** | **Age** | **Clade** | **Paper** | **Analysis** | **Coordinates** |
| --- | --- | --- | --- | --- | --- | --- | --- | --- |
| Bie6 | 65 | KX496902 | Poland | Modern | Western | Borowsky et al. (2016) | 4 |  |
| Bie9 | 65 | KX496903 | Poland | Modern | Western | Borowsky et al. (2016) | 4 |  |
| Stn2 | 9 | KX496904 | Poland | Modern | Western | Borowsky et al. (2016) | 4 |  |
| Stn9 | 65 | KX496905 | Poland | Modern | Western | Borowsky et al. (2016) | 4 |  |
| Tr10 | 9 | KX496906 | Poland | Modern | Western | Borowsky et al. (2016) | 4 |  |
| Gol1 | 25 | KX496907 | Poland | Modern | Western | Borowsky et al. (2016) | 4 |  |
| Gol2 | 31 | KX496908 | Poland | Modern | Western | Borowsky et al. (2016) | 4 |  |
| Gol3 | 9 | KX496909 | Poland | Modern | Western | Borowsky et al. (2016) | 4 |  |
| Gol8 | 66 | KX496910 | Poland | Modern | Western | Borowsky et al. (2016) | 4 |  |
| Bie1 | 5 | KX496911 | Poland | Modern | Western | Borowsky et al. (2016) | 4 |  |
| Rpo1 | 5 | KX496911 | Poland | Modern | Western | Borowsky et al. (2016) | 4 |  |
| Rpo3 | 9 | KX496912 | Poland | Modern | Western | Borowsky et al. (2016) | 4 |  |
| Rpo5 | 66 | KX496913 | Poland | Modern | Western | Borowsky et al. (2016) | 4 |  |
| Rpo7 | 28 | KX496914 | Poland | Modern | Western | Borowsky et al. (2016) | 4 |  |
| Stn4 | 65 | KX496915 | Poland | Modern | Western | Borowsky et al. (2016) | 4 |  |
| Sar1 | 148 | KX496916 | Poland | Modern | Western | Borowsky et al. (2016) | 4 |  |
| Sar3 | 149 | KX496917 | Poland | Modern | Western | Borowsky et al. (2016) | 4 |  |
| Mil7 | 21 | KX496918 | Poland | Modern | Western | Borowsky et al. (2016) | 4 |  |
| Brw1 | 66 | KX496919 | Poland | Modern | Western | Borowsky et al. (2016) | 4 |  |
| Gbr5 | 9 | KX496920 | Poland | Modern | Western | Borowsky et al. (2016) | 4 |  |
| Rus5 | 150 | KX496921 | Poland | Modern | Western | Borowsky et al. (2016) | 4 |  |
| Rud1 | 18 | KX496922 | Poland | Modern | Western | Borowsky et al. (2016) | 4 |  |
| Lbi4 | 28 | KX496923 | Poland | Modern | Western | Borowsky et al. (2016) | 4 |  |
| Lbi5 | 31 | KX496924 | Poland | Modern | Western | Borowsky et al. (2016) | 4 |  |
| Lut4 | 9 | KX496925 | Poland | Modern | Western | Borowsky et al. (2016) | 4 |  |
| Lut1 | 42 | KX496926 | Poland | Modern | Eastern | Borowsky et al. (2016) | 4 |  |
| Lut6 | 40 | KX496927 | Poland | Modern | Eastern | Borowsky et al. (2016) | 4 |  |
| Lad1 | 39 | KX496928 | Poland | Modern | Eastern | Borowsky et al. (2016) | 4 |  |
| Lad2 | 40 | KX496929 | Poland | Modern | Eastern | Borowsky et al. (2016) | 4 |  |
| Szk1 | 39 | KX496930 | Poland | Modern | Eastern | Borowsky et al. (2016) | 4 |  |
| Szk6 | 151 | KX496931 | Poland | Modern | Eastern | Borowsky et al. (2016) | 4 |  |
| Bal6 | 40 | KX496932 | Poland | Modern | Eastern | Borowsky et al. (2016) | 4 |  |
| Bir1 | 152 | KX496933 | Poland | Modern | Eastern | Borowsky et al. (2016) | 4 |  |
| Bir6 | 42 | KX496934 | Poland | Modern | Eastern | Borowsky et al. (2016) | 4 |  |
| Bal4 | 71 | KX496935 | Poland | Modern | Italy | Borowsky et al. (2016) | 4 |  |
| IRE1 | 14 | JQ599359 | Ireland | 2400 | Western | Carden et al. (2012) | 4 |  |
| IRE1 | 14 | JQ599359 | Ireland | modern | Western | Carden et al. (2012) | 4 |  |
| IRE2 | 14 | JQ599360 | Ireland | modern | Western | Carden et al. (2012) | 4 |  |
| IRE2 | 14 | JQ599360 | Ireland | 30000 | Western | Carden et al. (2012) | 4 |  |
| IRE3 | 13 | JQ599361 | Scotland | modern | Western | Carden et al. (2012) | 4 |  |
| IRE4 | 20 | JQ599362 | Scotland | modern | Western | Carden et al. (2012) | 4 |  |
| IRE5 | 23 | JQ599363 | Scotland | modern | Western | Carden et al. (2012) | 4 |  |
| IRE6 | 18 | JQ599364 | Spain | modern | Western | Carden et al. (2012) | 4 |  |
| IRE7 | 64 | JQ599365 | Scotland | modern | Western | Carden et al. (2012) | 4 |  |
| IRE8 | 19 | JQ599366 | Scotland | modern | Western | Carden et al. (2012) | 4 |  |
| IRE9 | 16 | JQ599367 | Scotland | modern | Western | Carden et al. (2012) | 4 |  |
| IRE10 | 21 | JQ599368 | Ireland | modern | Western | Carden et al. (2012) | 4 |  |
| IRE11 | 1 | JQ599369 | Ireland | modern | Western | Carden et al. (2012) | 4 |  |
| IRE12 | 1 | JQ599370 | Ireland | modern | Western | Carden et al. (2012) | 4 |  |
| IRE13 | 11 | JQ599371 | Ireland | modern | Italy | Carden et al. (2012) | 4 |  |
| IRE14 | 11 | JQ599372. | Scotland | modern | Italy | Carden et al. (2012) | 4 |  |
| IRE16 | 76 | JQ599374 | Ireland | 2789 | Western | Carden et al. (2012) | 4 |  |
| IRE16 | 76 | JQ599374. | ireland | 2900 | Western | Carden et al. (2012) | 4 |  |
| IRE17 | 77 | JQ599375 | Ireland | 1979 | Western | Carden et al. (2012) | 4 |  |
| IRE18 | 14 | JQ599376 | ireland | 1800 | Western | Carden et al. (2012) | 4 |  |
| UIST | 78 | JQ599377 | UK | modern | Western | Carden et al. (2012) | 4 |  |
| UIST | 78 | JQ599377 | UK | 2 | Western | Carden et al. (2012) | 4 |  |
| hap1 | 127 | KT202236.1 | Spain | modern | Western | Carranza et al. (2016) | 4 |  |
| hap2 | 128 | KT202237.1 | Spain | modern | Western | Carranza et al. (2016) | 4 |  |
| hap3 | 18 | KT202238.1 | Spain | modern | Western | Carranza et al. (2016) | 4 |  |
| hap4 | 129 | KT202239.1 | Spain | modern | Western | Carranza et al. (2016) | 4 |  |
| hap5 | 122 | KT202240.1 | Spain | modern | Western | Carranza et al. (2016) | 4 |  |
| hap6 | 21 | KT202241.1 | Spain | modern | Western | Carranza et al. (2016) | 4 |  |
| hap7 | 130 | KT202242.1 | Spain | modern | Western | Carranza et al. (2016) | 4 |  |
| hap8 | 131 | KT202243.1 | Spain | modern | Western | Carranza et al. (2016) | 4 |  |
| hap9 | 38 | KT202244.1 | Spain | modern | Western | Carranza et al. (2016) | 4 |  |
| hap10 | 37 | KT202245.1 | Spain | modern | Western | Carranza et al. (2016) | 4 |  |
| hap11 | 18 | KT202246.1 | Spain | modern | Western | Carranza et al. (2016) | 4 |  |
| hap12 | 36 | KT202247.1 | Spain | modern | Western | Carranza et al. (2016) | 4 |  |
| hap13 | 25 | KT202248.1 | Spain | modern | Western | Carranza et al. (2016) | 4 |  |
| hap14 | 132 | KT202249.1 | Spain | modern | Western | Carranza et al. (2016) | 4 |  |
| hap15 | 133 | KT202250.1 | Spain | modern | Western | Carranza et al. (2016) | 4 |  |
| hap16 | 18 | KT202251.1 | Spain | modern | Western | Carranza et al. (2016) | 4 |  |
| hap17 | 19 | KT202252.1 | Spain | modern | Western | Carranza et al. (2016) | 4 |  |
| hap18 | 20 | KT202253.1 | Spain | modern | Western | Carranza et al. (2016) | 4 |  |
| hap19 | 9 | KT202254.1 | Spain | modern | Western | Carranza et al. (2016) | 4 |  |
| hap20 | 9 | KT202255.1 | Spain | modern | Western | Carranza et al. (2016) | 4 |  |
| hap21 | 39 | KT202256.1 | Spain | modern | Eastern | Carranza et al. (2016) | 4 |  |
| hap22 | 13 | KT202257.1 | Spain | modern | Western | Carranza et al. (2016) | 4 |  |
| hap23 | 29 | KT202258.1 | Spain | modern | Western | Carranza et al. (2016) | 4 |  |
| hap24 | 14 | KT202259.1 | Spain | modern | Western | Carranza et al. (2016) | 4 |  |
| hap25 | 14 | KT202260.1 | Spain | modern | Western | Carranza et al. (2016) | 4 |  |
| hap26 | 39 | KT202261.1 | Spain | modern | Eastern | Carranza et al. (2016) | 4 |  |
| hap27 | 1 | KT202262.1 | Spain | modern | Western | Carranza et al. (2016) | 4 |  |
| hap28 | 9 | KT202263.1 | Spain | modern | Western | Carranza et al. (2016) | 4 |  |
| hap29 | 1 | KT202264.1 | Spain | modern | Western | Carranza et al. (2016) | 4 |  |
| hap30 | 18 | KT202265.1 | Spain | modern | Western | Carranza et al. (2016) | 4 |  |
| hap31 | 21 | KT202266.1 | Spain | modern | Western | Carranza et al. (2016) | 4 |  |
| hap32 | 27 | KT202267.1 | Spain | modern | Western | Carranza et al. (2016) | 4 |  |
| hap33 | 27 | KT202268.1 | Spain | modern | Western | Carranza et al. (2016) | 4 |  |
| hap34 | 40 | KT202269.1 | Spain | modern | Eastern | Carranza et al. (2016) | 4 |  |
| hap35 | 38 | KT202270.1 | Spain | modern | Western | Carranza et al. (2016) | 4 |  |
| hap36 | 1 | KT202271.1 | Spain | modern | Western | Carranza et al. (2016) | 4 |  |
| hap37 | 127 | KT202272.1 | Spain | modern | Western | Carranza et al. (2016) | 4 |  |
| hap38 | 22 | KT202273.1 | Spain | modern | Western | Carranza et al. (2016) | 4 |  |
| hap39 | 127 | KT202274.1 | Spain | modern | Western | Carranza et al. (2016) | 4 |  |
| hap40 | 37 | KT202275.1 | Spain | modern | Western | Carranza et al. (2016) | 4 |  |
| hap41 | 134 | KT202276.1 | Spain | modern | Western | Carranza et al. (2016) | 4 |  |
| hap42 | 135 | KT202277.1 | Spain | modern | Western | Carranza et al. (2016) | 4 |  |
| hap43 | 136 | KT202278.1 | Spain | modern | Western | Carranza et al. (2016) | 4 |  |
| hap44 | 21 | KT202279.1 | Spain | modern | Western | Carranza et al. (2016) | 4 |  |
| hap45 | 137 | KT202280.1 | Spain | modern | Western | Carranza et al. (2016) | 4 |  |
| HT02 | 9 | jq004399 | Czech Republic | modern | Western | Fickel et al. (2012) | 4 |  |
| HT04 | 46 | jq004400 | Czech Republic | modern | Western | Fickel et al. (2012) | 4 |  |
| HT04 | 46 | jq004400 | Germany | modern | Western | Fickel et al. (2012) | 4 |  |
| KT290948 | 42 | KT290948 | Hungary | modern | Eastern/Western | Frank et al. (2016) | 4 |  |
| NTysvHj1032 | 9 | gu369821 | Norway | modern | Western | Haanes et al. (2010) | 4 |  |
| NTysvHj1029 | 9 | gu369822 | Norway | modern | Western | Haanes et al. (2010) | 4 |  |
| NTysvHj1034 | 9 | gu369823 | Norway | modern | Western | Haanes et al. (2010) | 4 |  |
| NTysvHj1035 | 9 | gu369824 | Norway | modern | Western | Haanes et al. (2010) | 4 |  |
| N-Sund-Hj1088 | 1 | gu369825 | Norway | modern | Western | Haanes et al. (2010) | 4 |  |
| N-Sund-Hj1089 | 1 | gu369826 | Norway | modern | Western | Haanes et al. (2010) | 4 |  |
| N-Sund-Hj1090 | 18 | gu369827 | Norway | modern | Western | Haanes et al. (2010) | 4 |  |
| NSundHj1093 | 9 | gu369828 | Norway | modern | Western | Haanes et al. (2010) | 4 |  |
| NGAulHj1327 | 9 | gu369829 | Norway | modern | Western | Haanes et al. (2010) | 4 |  |
| NGAulHj1330 | 29 | gu369830 | Norway | modern | Western | Haanes et al. (2010) | 4 |  |
| NGAulHj1332 | 9 | gu369831 | Norway | modern | Western | Haanes et al. (2010) | 4 |  |
| NGAulHj1334 | 9 | gu369832 | Norway | modern | Western | Haanes et al. (2010) | 4 |  |
| N-Sund-Hj1091 | 1 | gu369833 | Norway | modern | Western | Haanes et al. (2010) | 4 |  |
| N-Sund-Hj1087 | 1 | gu369834 | Norway | modern | Western | Haanes et al. (2010) | 4 |  |
| NTysvHj1033 | 9 | gu369835 | Norway | modern | Western | Haanes et al. (2010) | 4 |  |
| NTysvHj1030 | 9 | gu369836 | Norway | modern | Western | Haanes et al. (2010) | 4 |  |
| NGAulHj1348 | 9 | gu369837 | Norway | modern | Western | Haanes et al. (2010) | 4 |  |
| N-OTTer-Hj1265 | 1 | gu369838 | Norway | modern | Western | Haanes et al. (2010) | 4 |  |
| N-OTTer-Hj1266 | 1 | gu369839 | Norway | modern | Western | Haanes et al. (2010) | 4 |  |
| NOTTerHj1268 | 31 | gu369840 | Norway | modern | Western | Haanes et al. (2010) | 4 |  |
| N-OTTer-Hj1282 | 1 | gu369841 | Norway | modern | Western | Haanes et al. (2010) | 4 |  |
| N-OTTer-Hj1288 | 1 | gu369842 | Norway | modern | Western | Haanes et al. (2010) | 4 |  |
| N-OTTer-Hj1291 | 1 | gu369843 | Norway | modern | Western | Haanes et al. (2010) | 4 |  |
| NOTTerHj1299 | 31 | gu369844 | Norway | modern | Western | Haanes et al. (2010) | 4 |  |
| NOTTerHj1301 | 31 | gu369845 | Norway | modern | Western | Haanes et al. (2010) | 4 |  |
| N-OTTer-Hj1337 | 1 | gu369846 | Norway | modern | Western | Haanes et al. (2010) | 4 |  |
| N-OTTer-Hj1341 | 1 | gu369847 | Norway | modern | Western | Haanes et al. (2010) | 4 |  |
| N-OTTer-Hj1347 | 1 | gu369848 | Norway | modern | Western | Haanes et al. (2010) | 4 |  |
| NOTTerHj1349 | 31 | gu369849 | Norway | modern | Western | Haanes et al. (2010) | 4 |  |
| NOTTer_Hj1353 | 31 | gu369850 | Norway | modern | Western | Haanes et al. (2010) | 4 |  |
| NOTTerHj1354 | 31 | gu369851 | Norway | modern | Western | Haanes et al. (2010) | 4 |  |
| N-OTTer-Hj1355 | 1 | gu369852 | Norway | modern | Western | Haanes et al. (2010) | 4 |  |
| NOTTerHj1356 | 31 | gu369853 | Norway | modern | Western | Haanes et al. (2010) | 4 |  |
| HunHj1678 | 47 | gu369854 | Hungary | modern | Eastern | Haanes et al. (2010) | 4 |  |
| HunHj1679 | 47 | gu369855 | Hungary | modern | Eastern | Haanes et al. (2010) | 4 |  |
| HunHj1667 | 47 | gu369856 | Hungary | modern | Eastern | Haanes et al. (2010) | 4 |  |
| HunHj1680 | 65 | gu369857 | Hungary | modern | Western | Haanes et al. (2010) | 4 |  |
| HunHj1669 | 47 | gu369858 | Hungary | modern | Eastern | Haanes et al. (2010) | 4 |  |
| HunHj1670 | 42 | gu369859 | Hungary | modern | Eastern | Haanes et al. (2010) | 4 |  |
| HunHj1671 | 47 | gu369860 | Hungary | modern | Eastern | Haanes et al. (2010) | 4 |  |
| HunHj1672 | 42 | gu369861 | Hungary | modern | Eastern | Haanes et al. (2010) | 4 |  |
| HunHj1673 | 42 | gu369862 | Hungary | modern | Eastern | Haanes et al. (2010) | 4 |  |
| HunHj1674 | 47 | gu369863 | Hungary | modern | Eastern | Haanes et al. (2010) | 4 |  |
| HunHj1676 | 47 | gu369864 | Hungary | modern | Eastern | Haanes et al. (2010) | 4 |  |
| HunHj1677 | 47 | gu369865 | Hungary | modern | Eastern | Haanes et al. (2010) | 4 |  |
| HunHj1668 | 47 | gu369866 | Hungary | modern | Eastern | Haanes et al. (2010) | 4 |  |
| HunHj1661 | 47 | gu369867 | Hungary | modern | Eastern | Haanes et al. (2010) | 4 |  |
| HunHj1662 | 47 | gu369868 | Hungary | modern | Eastern | Haanes et al. (2010) | 4 |  |
| HunHj1663 | 47 | gu369869 | Hungary | modern | Eastern | Haanes et al. (2010) | 4 |  |
| HunHj1664 | 47 | gu369870 | Hungary | modern | Eastern | Haanes et al. (2010) | 4 |  |
| HunHj1665 | 47 | gu369871 | Hungary | modern | Eastern | Haanes et al. (2010) | 4 |  |
| Hmwe01 | 15 | DQ452075 | UK | modern | Western | Hmwe et al. (2006) | 4 |  |
| Hmwe02 | 14 | DQ452076 | UK | modern | Western | Hmwe et al. (2006) | 4 |  |
| Hmwe03 | 16 | DQ452077 | UK | modern | Western | Hmwe et al. (2006) | 4 |  |
| Hmwe04 | 13 | DQ452078 | UK | modern | Western | Hmwe et al. (2006) | 4 |  |
| Hmwe05 | 17 | DQ452079 | UK | modern | Western | Hmwe et al. (2006) | 4 |  |
| Hmwe06 | 1 | DQ452080 | UK | modern | Western | Hmwe et al. (2006) | 4 |  |
| Hmwe07 | 1 | DQ452081 | UK | modern | Western | Hmwe et al. (2006) | 4 |  |
| Hmwe08 | 18 | DQ452082 | UK | modern | Western | Hmwe et al. (2006) | 4 |  |
| Hmwe09 | 19 | DQ452083 | UK | modern | Western | Hmwe et al. (2006) | 4 |  |
| Hmwe10 | 9 | DQ452084 | UK | modern | Western | Hmwe et al. (2006) | 4 |  |
| Hmwe11 | 20 | DQ452085 | UK | modern | Western | Hmwe et al. (2006) | 4 |  |
| Hmwe12 | 15 | DQ452086 | UK | modern | Western | Hmwe et al. (2006) | 4 |  |
| Hmwe13 | 1 | DQ452087 | UK | modern | Western | Hmwe et al. (2006) | 4 |  |
| NW1 | 13 | fj743483 | Ireland | modern | Western | McDevitt et al. (2009) | 4 |  |
| NW2 | 1 | fj743484 | Ireland | modern | Western | McDevitt et al. (2009) | 4 |  |
| NW3 | 23 | fj743485 | Ireland | modern | Western | McDevitt et al. (2009) | 4 |  |
| KNP1 | 14 | fj743486 | Ireland | modern | Western | McDevitt et al. (2009) | 4 |  |
| KNP2 | 14 | fj743487 | Ireland | modern | Western | McDevitt et al. (2009) | 4 |  |
| KNP3 | 62 | fj743488 | Ireland | modern | Western | McDevitt et al. (2009) | 4 |  |
| KNP4 | 14 | fj743489 | Ireland | modern | Western | McDevitt et al. (2009) | 4 |  |
| KNP5 | 14 | fj743490 | Ireland | modern | Western | McDevitt et al. (2009) | 4 |  |
| KNP6 | 63 | fj743491 | Ireland | modern | Western | McDevitt et al. (2009) | 4 |  |
| KNP7 | 14 | fj743492 | Ireland | modern | Western | McDevitt et al. (2009) | 4 |  |
| SCB1 | 64 | fj743493 | Ireland | modern | Western | McDevitt et al. (2009) | 4 |  |
| SCB2 | 16 | fj743494 | Ireland | modern | Western | McDevitt et al. (2009) | 4 |  |
| SCB3 | 1 | fj743495 | Ireland | modern | Western | McDevitt et al. (2009) | 4 |  |
| SCB4 | 21 | fj743496 | Ireland | modern | Western | McDevitt et al. (2009) | 4 |  |
| MY1 | 19 | fj743497 | Ireland | modern | Western | McDevitt et al. (2009) | 4 |  |
| WK1 | 11 | fj743498 | Ireland | modern | Western | McDevitt et al. (2009) | 4 |  |
| WK2 | 1 | fj743499 | Ireland | modern | Western | McDevitt et al. (2009) | 4 |  |
| MM007 | 82 | KF133903 | Spain, El Mirón Cave | Lower Magdalenian | Western | Meiri et al. (2013) | 1,2,3,4 | 50.4675,-3.5025 |
| MM020 | 83 | KF133904 | Caucasus, North Ossetia, Cave Lasok | 32,040±180 | Eastern | Meiri et al. (2013) | 1,2,3,4 | 43.2339,44.0597 |
| MM079 | 84 | KF133905 | Spain, El Mirón Cave | 11,785±55 | Western | Meiri et al. (2013) | 1,2,3,4,5 | 50.4675,-3.5025 |
| MM080 | 85 | KF133906 | Spain, El Mirón Cave | 11,205±55 | Western | Meiri et al. (2013) | 1,2,3,4,5 | 50.4675,-3.5025 |
| MM082 | 86 | KF133907 | Spain, El Mirón Cave | 14,930±70 | Eastern | Meiri et al. (2013) | 1,2,3,4,5 | 50.4675,-3.5025 |
| MM093 | 86 | KF133908 | Spain, El Mirón Cave | 14,760±70, 14,795±75 | Eastern | Meiri et al. (2013) | 1,2,3,4,5 | 50.4675,-3.5025 |
| MM095 | 87 | KF133909 | Spain, El Mirón Cave | Magdalenian | Western | Meiri et al. (2013) | 1,2,3,4,5 | 50.4675,-3.5025 |
| MM098 | 88 | KF133910 | Spain, El Mirón Cave | 15,430±75 | Eastern | Meiri et al. (2013) | 1,2,3,4,5 | 50.4675,-3.5025 |
| MM131 | 89 | KF133911 | England, Elder Bush Cave | 10,600±110 | Western | Meiri et al. (2013) | 1,2,3,4 | 53.093668, -1.832575 |
| MM135 | 90 | KF133912 | France, Le Bois Ragot | 12,585±75 | Western | Meiri et al. (2013) | 1,2,3,4 | 46.366449, 0.680459 |
| MM139 | 91 | KF133913 | France, Prissé-la-Charrière | 5460±45 | Western | Meiri et al. (2013) | 1,2,3,4 | 46.150923, -0.483282 |
| MM150 | 92 | KF133914 | England, Longstones Field | 4193±35 | Western | Meiri et al. (2013) | 1,2,3,4 | 51.428786, -1.855170 |
| MM153 | 93 | KF133915 | Serbia, Rudna Glava S7 | 7198±36 | Eastern | Meiri et al. (2013) | 1,2,3,4 | 44.339738, 22.034960 |
| MM154 | 94 | KF133916 | England, Longstones Field | 4216±36 | Western | Meiri et al. (2013) | 1,2,3,4 | 51.428786, -1.855170 |
| MM165 | 95 | KF133917 | Serbia, Lepenski Vir S47 | 10,000±45 | Eastern | Meiri et al. (2013) | 1,2,3,4 | 44.552714, 22.027393 |
| MM175 | 96 | KF133918 | England, Hyena Den, Wookey Hole | 11,320±120 | Western | Meiri et al. (2013) | 1,2,3,4 | 51.226390, -2.672081 |
| MM183 | 97 | KF133919 | Scotland, Isle of Risga | 5875±65 | Western | Meiri et al. (2013) | 1,2,3,4 | 56.668623, -5.908740 |
| MM186 | 89 | KF133920 | Scotland, Shewalton | 5840±80 | Western | Meiri et al. (2013) | 1,2,3,4 | 55.595770, -4.605892 |
| MM187 | 98 | KF133921 | Spain, El Mirón Cave | 15,610±90 | Western | Meiri et al. (2013) | 1,2,3,4,5 | 50.4675,-3.5025 |
| MM190 | 99 | KF133922 | Serbia, Lepenski Vir S46 | 7912±39 | Western | Meiri et al. (2013) | 1,2,3,4 | 44.886046, 12.239328 |
| MM192 | 100 | KF133923 | Caucasus, Mezmaiska Cave | >44,700 | Western | Meiri et al. (2013) | 1,2,3,4 | 44.0994,39.9222 |
| MM198 | 95 | KF133924 | England,+A1:I436 | 12,310±50 | Eastern | Meiri et al. (2013) | 1,2,3,4 | 52.808474, -2.011795 |
| MM210 | 101 | KF133925 | North Sea | 8870±50 | Western | Meiri et al. (2013) | 1,2,3,4 | 56.942428, 4.277717 |
| MM214 | 102 | KF133926 | Caucasus, Mezmaiska Cave | 50,100±4000 | Eastern | Meiri et al. (2013) | 1,2,3,4 | 44.0994,39.9222 |
| MM224 | 103 | KF133927 | Caucasus, Mezmaiska Cave | >50,200 | Eastern | Meiri et al. (2013) | 1,2,3,4 | 44.0994,39.9222 |
| MM235 | 104 | KF133928 | Belgium, Trou Al'Wesse Couche 4a | Neolithic | Western | Meiri et al. (2013) | 1,2,3,4 | 50.421, 5.294 |
| MM239 | 105 | KF133929 | Belgium, Trou Al'Wesse Couche 4b | Mesolithic | Western | Meiri et al. (2013) | 1,2,3,4 | 50.421, 5.294 |
| MM245 | 106 | KF133930 | Belgium, Trou Al'Wesse Couche 15 | 40,200±1300 | Eastern | Meiri et al. (2013) | 1,2,3,4 | 50.421, 5.294 |
| MM251 | 107 | KF133931 | England, Chelm's Combe, Cheddar | 10,910±110 | Western | Meiri et al. (2013) | 1,2,3,4 | 51.280893, -2.778675 |
| MM272 | 103 | KF133932 | Urals | 44,650±650 | Eastern | Meiri et al. (2013) | 1,2,3,4 | 60.367639, 58.366680 |
| MM352 | 107 | KF133933 | Caucasus, Mezmaiska Cave | pre-LGM: between ca. 60,000 and 25,000 cal. yr BP | Western | Meiri et al. (2013) | 1,2,3,4 | 44.0994,39.9222 |
| MM379 | 109 | KF133934 | Urals | >45,500 | Eastern | Meiri et al. (2013) | 1,2,3,4 | 60.367639, 58.366680 |
| MM408 | 110 | KF133935 | Jordan, Tell Hesban | 864±28 | Eastern | Meiri et al. (2013) | 1,2,3,4 | 31.715606, 35.806639 |
| MM415 | 111 | KF133936 | Jordan, Tell Hesban | 864±28 | Eastern | Meiri et al. (2013) | 1,2,3,4 | 31.715606, 35.806639 |
| MM455 | 112 | KF133937 | England, Kent's Cavern | pre-LGM: between ca. 60,000 and 25,000 cal. yr BP | Eastern | Meiri et al. (2013) | 1,2,3,4 | 50.4675,-3.5025 |
| MM461 | 113 | KF133938 | Spain, Mazaculos Shelter | Neolithic | Western | Meiri et al. (2013) | 1,2,3,4,5 | 43.387233, -4.572220 |
| MM464 | 114 | KF133939 | Spain, Mazaculos Shelter | Neolithic | Western | Meiri et al. (2013) | 1,2,3,4,5 | 43.387233, -4.572220 |
| MM472 | 115 | KF133940 | Spain, Mazaculos Shelter | Neolithic | Western | Meiri et al. (2013) | 1,2,3,4,5 | 43.387233, -4.572220 |
| MM473 | 116 | KF133941 | Germany, Rosenbeck | Ancient | Western | Meiri et al. (2013) | 1,2,4 |  |
| MM475 | 117 | KF133942 | Spain, Mazaculos Shelter | Austurian | Western | Meiri et al. (2013) | 1,2,3,4,5 | 43.387233, -4.572220 |
| MM468 | 118 | KF133943 | Spain, Mazaculos Shelter | Neolithic | Western | Meiri et al. (2013) | 1,2,3,4,5 | 43.387233, -4.572220 |
| MM477 | 107 | KF133944 | Spain, Mazaculos Shelter | Austurian | Western | Meiri et al. (2013) | 1,2,3,4,5 | 43.387233, -4.572220 |
| MM493 | 119 | KF133945 | Spain, Mazaculos Shelter | Austurian | Western | Meiri et al. (2013) | 1,2,3,4,5 | 43.387233, -4.572220 |
| MM762 | 9 | KF133946 | Norway, Etne | Modern | Western | Meiri et al. (2013) | 1,2,4 |  |
| MM763 | 9 | KF133955 | Norway, Etne | Modern | Western | Meiri et al. (2013) | 1,2,4 |  |
| MM764 | 9 | KF133956 | Norway, Etne | Modern | Western | Meiri et al. (2013) | 1,2,4 |  |
| MM702 | 120 | KF133947 | Czech Republic, Homolka Pit 31a | Modern | Western | Meiri et al. (2013) | 1,2,4 |  |
| MM335 | 66 | KF133949 | Belarus, Belovezhskaya Pushcha Reserve | Modern | Western | Meiri et al. (2013) | 1,2,4 |  |
| MM337 | 66 | KF133950 | Russia, Karelia | Modern | Western | Meiri et al. (2013) | 1,2,4 |  |
| MM719 | 74 | KF133951 | Germany, Hamburg | Modern | Western | Meiri et al. (2013) | 1,2,4 |  |
| MM754 | 39 | KF133952 | Italy, Southern Apennine Mountains, Molise | Modern | Eastern | Meiri et al. (2013) | 1,2,4 |  |
| MM755 | 39 | KF133953 | Italy, Southern Apennine Mountains, Molise | Modern | Eastern | Meiri et al. (2013) | 1,2,4 |  |
| MM770 | 121 | KF133954 | Sweden, Skane | Modern | Western | Meiri et al. (2013) | 1,2,4 |  |
| MM765 | 9 | KF133957 | Norway, Sveio | Modern | Western | Meiri et al. (2013) | 1,2,4 |  |
| MM721 | 122 | KF133958 | Spain, Coto Doñana Huelua | Modern | Western | Meiri et al. (2013) | 1,2,4 |  |
| MM731 | 122 | KF133959 | Spain, Coto Doñana Huelua | Modern | Western | Meiri et al. (2013) | 1,2,4 |  |
| MM718 | 25 | KF133960 | Spain, Castilla-La Mancha | Modern | Western | Meiri et al. (2013) | 1,2,4 |  |
| MM781 | 30 | KF133961 | Italy, Mesola Wood (Gran Bosco della Mesola) | Modern | Western | Meiri et al. (2013) | 1,2,4 |  |
| MM782 | 30 | KF133962 | Italy, Mesola Wood (Gran Bosco della Mesola) | Modern | Western | Meiri et al. (2013) | 1,2,4 |  |
| MM313 | 68 | KF133963 | Russia, Voronezh | Modern | Western | Meiri et al. (2013) | 1,2,4 |  |
| MM334 | 68 | KF133948 | Russia, Voronezh | Modern | Western | Meiri et al. (2013) | 1,2,4 |  |
| MM315 | 123 | KF133964 | Russia, Krasnodarsky Krai, Caucasus | Modern | Eastern | Meiri et al. (2013) | 1,2,4 |  |
| MM317 | 123 | KF133965 | Russia, Krasnodarsky Krai, Caucasus | Modern | Eastern | Meiri et al. (2013) | 1,2,4 |  |
| MM766 | 124 | KF133966 | Turkey, Central Anatolia | Modern | Eastern | Meiri et al. (2013) | 1,2,4 |  |
| MM331 | 125 | KF133967 | Georgia, Borzon | Modern | Eastern | Meiri et al. (2013) | 1,2,4 |  |
| MM732 | 23 | KF133968 | Scotland, Inverness- Shire Glenquoich | Modern | Western | Meiri et al. (2013) | 1,2,4 |  |
| MM733 | 23 | KF133969 | Scotland | Modern | Western | Meiri et al. (2013) | 1,2,4 |  |
| hap1 | 65 | HQ290005.1 | Germany | modern | Western | Niedziałkowska et al. (2011) | 4 |  |
| hap2 | 25 | HQ290006.1 | Germany | modern | Western | Niedziałkowska et al. (2011) | 4 |  |
| hap3 | 9 | HQ290007.1 | Poland | modern | Western | Niedziałkowska et al. (2011) | 4 |  |
| hap4 | 66 | HQ290008.1 | Belarus | modern | Western | Niedziałkowska et al. (2011) | 4 |  |
| hap5 | 18 | HQ290009.1 | Poland | modern | Western | Niedziałkowska et al. (2011) | 4 |  |
| hap6 | 67 | HQ290010.1 | Poland | modern | Western | Niedziałkowska et al. (2011) | 4 |  |
| hap7 | 42 | HQ290011.1 | Ukraine | modern | Eastern | Niedziałkowska et al. (2011) | 4 |  |
| hap8 | 9 | HQ290012.1 | Germany | modern | Western | Niedziałkowska et al. (2011) | 4 |  |
| hap9 | 68 | HQ290013.1 | Belarus | modern | Eastern | Niedziałkowska et al. (2011) | 4 |  |
| hap10 | 9 | HQ290014.1 | Germany | modern | Western | Niedziałkowska et al. (2011) | 4 |  |
| hap11 | 39 | HQ290015.1 | Poland | modern | Eastern | Niedziałkowska et al. (2011) | 4 |  |
| hap12 | 47 | HQ290016.1 | Belarus | modern | Eastern | Niedziałkowska et al. (2011) | 4 |  |
| hap13 | 40 | HQ290017.1 | Poland | modern | Eastern | Niedziałkowska et al. (2011) | 4 |  |
| hap14 | 69 | HQ290018.1 | Germany | modern | Western | Niedziałkowska et al. (2011) | 4 |  |
| hap15 | 70 | HQ290019.1 | Belarus | modern | Eastern | Niedziałkowska et al. (2011) | 4 |  |
| hap16 | 154 | HQ290020.1 | Germany | modern | Western | Niedziałkowska et al. (2011) | 4 |  |
| hap17 | 1 | HQ290021.1 | Germany | modern | Western | Niedziałkowska et al. (2011) | 4 |  |
| hap18 | 23 | HQ290022.1 | Poland | modern | Eastern | Niedziałkowska et al. (2011) | 4 |  |
| hap19 | 40 | HQ290023.1 | Poland | modern | Eastern | Niedziałkowska et al. (2011) | 4 |  |
| hap20 | 71 | HQ290024.1 | Germany | modern | Western | Niedziałkowska et al. (2011) | 4 |  |
| hap21 | 21 | HQ290025.1 | France | modern | Western | Niedziałkowska et al. (2011) | 4 |  |
| hap22 | 22 | HQ290026.1 | France | modern | Western | Niedziałkowska et al. (2011) | 4 |  |
| hap23 | 23 | HQ290027.1 | France | modern | Western | Niedziałkowska et al. (2011) | 4 |  |
| hap24 | 16 | HQ290028.1 | France | modern | Western | Niedziałkowska et al. (2011) | 4 |  |
| hap25 | 18 | HQ290029.1 | Spain | modern | Western | Niedziałkowska et al. (2011) | 4 |  |
| hap26 | 24 | HQ290030.1 | Romania | modern | Western | Niedziałkowska et al. (2011) | 4 |  |
| hap27 | 25 | HQ290031.1 | Spain | modern | Western | Niedziałkowska et al. (2011) | 4 |  |
| hap28 | 26 | HQ290032.1 | Spain | modern | Western | Niedziałkowska et al. (2011) | 4 |  |
| hap29 | 1 | HQ290033.1 | scotland | modern | Western | Niedziałkowska et al. (2011) | 4 |  |
| hap30 | 27 | HQ290034.1 | Germany | modern | Western | Niedziałkowska et al. (2011) | 4 |  |
| hap31 | 27 | HQ290035.1 | Germany | modern | Western | Niedziałkowska et al. (2011) | 4 |  |
| hap32 | 19 | HQ290036.1 | Scotland | modern | Western | Niedziałkowska et al. (2011) | 4 |  |
| hap33 | 9 | HQ290037.1 | Scotland | modern | Western | Niedziałkowska et al. (2011) | 4 |  |
| hap34 | 27 | HQ290038.1 | Germany | modern | Western | Niedziałkowska et al. (2011) | 4 |  |
| hap35 | 1 | HQ290039.1 | Scotland | modern | Western | Niedziałkowska et al. (2011) | 4 |  |
| hap36 | 20 | HQ290040.1 | Scotland | modern | Western | Niedziałkowska et al. (2011) | 4 |  |
| hap37 | 28 | HQ290041.1 | Sweden | modern | Western | Niedziałkowska et al. (2011) | 4 |  |
| hap38 | 9 | HQ290042.1 | Norway | modern | Western | Niedziałkowska et al. (2011) | 4 |  |
| hap39 | 1 | HQ290043.1 | France | modern | Western | Niedziałkowska et al. (2011) | 4 |  |
| hap40 | 29 | HQ290044.1 | Norway | modern | Western | Niedziałkowska et al. (2011) | 4 |  |
| hap41 | 30 | HQ290045.1 | Italy | modern | Italy | Niedziałkowska et al. (2011) | 4 |  |
| hap42 | 31 | HQ290046.1 | Italy | modern | Western | Niedziałkowska et al. (2011) | 4 |  |
| hap43 | 32 | HQ290047.1 | Spain | modern | Western | Niedziałkowska et al. (2011) | 4 |  |
| hap44 | 33 | HQ290048.1 | Italy | modern | Western | Niedziałkowska et al. (2011) | 4 |  |
| hap45 | 34 | HQ290049.1 | Spain | modern | Western | Niedziałkowska et al. (2011) | 4 |  |
| hap46 | 35 | HQ290050.1 | Spain | modern | Western | Niedziałkowska et al. (2011) | 4 |  |
| hap47 | 36 | HQ290051.1 | Spain | modern | Western | Niedziałkowska et al. (2011) | 4 |  |
| hap48 | 37 | HQ290052.1 | Spain | modern | Western | Niedziałkowska et al. (2011) | 4 |  |
| hap49 | 38 | HQ290053.1 | Spain | modern | Western | Niedziałkowska et al. (2011) | 4 |  |
| hap50 | 41 | HQ290054.1 | Romania | modern | Eastern | Niedziałkowska et al. (2011) | 4 |  |
| hap51 | 43 | HQ290055.1 | Hungary | modern | Eastern | Niedziałkowska et al. (2011) | 4 |  |
| hap52 | 44 | HQ290056.1 | Bulgary | modern | Eastern | Niedziałkowska et al. (2011) | 4 |  |
| hap53 | 45 | HQ290057.1 | Italy | modern | Eastern | Niedziałkowska et al. (2011) | 4 |  |
| hap54 | 46 | HQ290058.1 | Romania | modern | Eastern | Niedziałkowska et al. (2011) | 4 |  |
| hap55 | 48 | HQ290059.1 | Romania | modern | Eastern | Niedziałkowska et al. (2011) | 4 |  |
| hap56 | 49 | HQ290060.1 | Italy | modern | Italy | Niedziałkowska et al. (2011) | 4 |  |
| hap57 | 50 | HQ290061.1 | Italy | modern | Italy | Niedziałkowska et al. (2011) | 4 |  |
| hap58 | 49 | HQ290062.1 | Italy | modern | Italy | Niedziałkowska et al. (2011) | 4 |  |
| hap59 | 11 | HQ290063.1 | Italy | modern | Italy | Niedziałkowska et al. (2011) | 4 |  |
| hap60 | 11 | HQ290064.1 | Italy | modern | Italy | Niedziałkowska et al. (2011) | 4 |  |
| hap61 | 72 | HQ290065.1 | Algeria | modern | Italy | Niedziałkowska et al. (2011) | 4 |  |
| hap62 | 59 | HQ290066.1 | Denmark | modern | Western | Niedziałkowska et al. (2011) | 4 |  |
| hap63 | 73 | HQ290067.1 | Germany | modern | Western | Niedziałkowska et al. (2011) | 4 |  |
| hap64 | 74 | HQ290068.1 | Germany | modern | Western | Niedziałkowska et al. (2011) | 4 |  |
| hap65 | 18 | HQ290069.1 | Germany | modern | Western | Niedziałkowska et al. (2011) | 4 |  |
| hap66 | 31 | HQ290070.1 | Germany | modern | Western | Niedziałkowska et al. (2011) | 4 |  |
| hap67 | 75 | HQ290071.1 | Poland | modern | Eastern | Niedziałkowska et al. (2011) | 4 |  |
| hap68 | 71 | HQ290072.1 | Poland | modern | Eastern | Niedziałkowska et al. (2011) | 4 |  |
| hap69 | 28 | HQ290073.1 | Germany | modern | Western | Niedziałkowska et al. (2011) | 4 |  |
| H1 | 59 | EU544179.1 | Denmark | modern | Western | Nielsen et al. (2008) | 4 |  |
| H2 | 23 | EU544180.1 | Denmark | modern | Western | Nielsen et al. (2008) | 4 |  |
| H3 | 28 | EU544181.1 | Denmark | modern | Western | Nielsen et al. (2008) | 4 |  |
| H4 | 42 | EU544182.1 | Denmark | modern | Western | Nielsen et al. (2008) | 4 |  |
| H5 | 59 | EU544183.1 | Denmark | modern | Western | Nielsen et al. (2008) | 4 |  |
| H6 | 60 | EU544184.1 | Denmark | modern | Western | Nielsen et al. (2008) | 4 |  |
| H7 | 61 | EU544185.1 | Denmark | modern | Western | Nielsen et al. (2008) | 4 |  |
| RUM A | 11 | DQ386106 | RUM (Scotland) | modern | Western | Nussey et al. (2006) | 4 |  |
| RUM B1 | 12 | DQ386107 | RUM (Scotland) | modern | Western | Nussey et al. (2006) | 4 |  |
| RUM B2 | 1 | DQ386108 | RUM (Scotland) | modern | Western | Nussey et al. (2006) | 4 |  |
| RUM B3 | 13 | DQ386109 | RUM(Scotland) | modern | Western | Nussey et al. (2006) | 4 |  |
| RUM B4 | 14 | DQ386110 | RUM(Scotland) | modern | Western | Nussey et al. (2006) | 4 |  |
| AB926017 | 1 | AB926017 | Tyrolean | 5100 | Western | Olivieri et al. (2014) | 4 |  |
| H1 | 14 | EF636727.1 | Highlands (Scotland) | modern | Western | Pérez-Espona et al. (2009) | 4 |  |
| H2 | 9 | EF636728.1 | Highlands (Scotland) | modern | Western | Pérez-Espona et al. (2009) | 4 |  |
| H3 | 14 | EF636729.1 | Highlands (Scotland) | modern | Western | Pérez-Espona et al. (2009) | 4 |  |
| H4 | 18 | EF636730.1 | Highlands (Scotland) | modern | Western | Pérez-Espona et al. (2009) | 4 |  |
| H5 | 9 | EF636731.1 | Highlands (Scotland) | modern | Western | Pérez-Espona et al. (2009) | 4 |  |
| H6 | 18 | EF636732.1 | Highlands (Scotland) | modern | Western | Pérez-Espona et al. (2009) | 4 |  |
| H7 | 9 | EF636733.1 | Highlands (Scotland) | modern | Western | Pérez-Espona et al. (2009) | 4 |  |
| H8 | 9 | EF636734.1 | Highlands (Scotland) | modern | Western | Pérez-Espona et al. (2009) | 4 |  |
| H9 | 23 | EF636735.1 | Highlands (Scotland) | modern | Western | Pérez-Espona et al. (2009) | 4 |  |
| H10 | 9 | EF636736.1 | Highlands (Scotland) | modern | Western | Pérez-Espona et al. (2009) | 4 |  |
| H11 | 1 | EF636737.1 | Highlands (Scotland) | modern | Western | Pérez-Espona et al. (2009) | 4 |  |
| H12 | 51 | EF636738.1 | Highlands (Scotland) | modern | Western | Pérez-Espona et al. (2009) | 4 |  |
| H13 | 52 | EF636739.1 | Highlands (Scotland) | modern | Western | Pérez-Espona et al. (2009) | 4 |  |
| H14 | 18 | EF636740.1 | Highlands (Scotland) | modern | Western | Pérez-Espona et al. (2009) | 4 |  |
| H15 | 14 | EF636741.1 | Highlands (Scotland) | modern | Western | Pérez-Espona et al. (2009) | 4 |  |
| H16 | 13 | EF636742.1 | Highlands (Scotland) | modern | Western | Pérez-Espona et al. (2009) | 4 |  |
| H17 | 1 | EF636743.1 | Highlands (Scotland) | modern | Western | Pérez-Espona et al. (2009) | 4 |  |
| H18 | 16 | EF636744.1 | Highlands (Scotland) | modern | Western | Pérez-Espona et al. (2009) | 4 |  |
| H19 | 19 | EF636745.1 | Highlands (Scotland) | modern | Western | Pérez-Espona et al. (2009) | 4 |  |
| H20 | 53 | EF636746.1 | Highlands (Scotland) | modern | Western | Pérez-Espona et al. (2009) | 4 |  |
| H21 | 20 | EF636747.1 | Highlands (Scotland) | modern | Western | Pérez-Espona et al. (2009) | 4 |  |
| H22 | 1 | EF636748.1 | Highlands (Scotland) | modern | Western | Pérez-Espona et al. (2009) | 4 |  |
| H23 | 18 | EF636749.1 | Highlands (Scotland) | modern | Western | Pérez-Espona et al. (2009) | 4 |  |
| H24 | 1 | EF636750.1 | Highlands (Scotland) | modern | Western | Pérez-Espona et al. (2009) | 4 |  |
| H25 | 1 | EF636751.1 | Highlands (Scotland) | modern | Western | Pérez-Espona et al. (2009) | 4 |  |
| H26 | 23 | EF636752.1 | Highlands (Scotland) | modern | Western | Pérez-Espona et al. (2009) | 4 |  |
| H27 | 9 | EF636753.1 | Highlands (Scotland) | modern | Western | Pérez-Espona et al. (2009) | 4 |  |
| H28 | 18 | EF636754.1 | Highlands (Scotland) | modern | Western | Pérez-Espona et al. (2009) | 4 |  |
| H29 | 14 | EF636755.1 | Highlands (Scotland) | modern | Western | Pérez-Espona et al. (2009) | 4 |  |
| H30 | 54 | EF636756.1 | Highlands (Scotland) | modern | Western | Pérez-Espona et al. (2009) | 4 |  |
| H31 | 55 | EF636757.1 | Highlands (Scotland) | modern | Western | Pérez-Espona et al. (2009) | 4 |  |
| H32 | 9 | EF636758.1 | Highlands (Scotland) | modern | Western | Pérez-Espona et al. (2009) | 4 |  |
| H33 | 18 | EF636759.1 | Highlands (Scotland) | modern | Western | Pérez-Espona et al. (2009) | 4 |  |
| H34 | 9 | EF636760.1 | Highlands (Scotland) | modern | Western | Pérez-Espona et al. (2009) | 4 |  |
| H35 | 9 | EF636761.1 | Highlands (Scotland) | modern | Western | Pérez-Espona et al. (2009) | 4 |  |
| H36 | 9 | EF636762.1 | Highlands (Scotland) | modern | Western | Pérez-Espona et al. (2009) | 4 |  |
| H37 | 56 | EF636763.1 | Highlands (Scotland) | modern | Western | Pérez-Espona et al. (2009) | 4 |  |
| H38 | 18 | EF636764.1 | Highlands (Scotland) | modern | Western | Pérez-Espona et al. (2009) | 4 |  |
| H39 | 18 | EF636765.1 | Highlands (Scotland) | modern | Western | Pérez-Espona et al. (2009) | 4 |  |
| H40 | 14 | EF636766.1 | Highlands (Scotland) | modern | Western | Pérez-Espona et al. (2009) | 4 |  |
| H41 | 9 | EF636767.1 | Highlands (Scotland) | modern | Western | Pérez-Espona et al. (2009) | 4 |  |
| H42 | 1 | EF636768.1 | Highlands (Scotland) | modern | Western | Pérez-Espona et al. (2009) | 4 |  |
| H43 | 57 | EF636769.1 | Highlands (Scotland) | modern | Western | Pérez-Espona et al. (2009) | 4 |  |
| H44 | 14 | EF636770.1 | Highlands (Scotland) | modern | Western | Pérez-Espona et al. (2009) | 4 |  |
| H45 | 9 | EF636771.1 | Highlands (Scotland) | modern | Western | Pérez-Espona et al. (2009) | 4 |  |
| H46 | 57 | EF636772.1 | Highlands (Scotland) | modern | Western | Pérez-Espona et al. (2009) | 4 |  |
| H47 | 9 | EF636773.1 | Highlands (Scotland) | modern | Western | Pérez-Espona et al. (2009) | 4 |  |
| H48 | 9 | EF636774.1 | Highlands (Scotland) | modern | Western | Pérez-Espona et al. (2009) | 4 |  |
| H49 | 9 | EF636775.1 | Highlands (Scotland) | modern | Western | Pérez-Espona et al. (2009) | 4 |  |
| H50 | 9 | EF636776.1 | Highlands (Scotland) | modern | Western | Pérez-Espona et al. (2009) | 4 |  |
| H51 | 19 | EF636777.1 | Highlands (Scotland) | modern | Western | Pérez-Espona et al. (2009) | 4 |  |
| H52 | 19 | EF636778.1 | Highlands (Scotland) | modern | Western | Pérez-Espona et al. (2009) | 4 |  |
| H53 | 1 | EF636779.1 | Highlands (Scotland) | modern | Western | Pérez-Espona et al. (2009) | 4 |  |
| H54 | 20 | EF636780.1 | Highlands (Scotland) | modern | Western | Pérez-Espona et al. (2009) | 4 |  |
| H55 | 58 | EF636781.1 | Highlands (Scotland) | modern | Western | Pérez-Espona et al. (2009) | 4 |  |
| H56 | 1 | EF636782.1 | Highlands (Scotland) | modern | Western | Pérez-Espona et al. (2009) | 4 |  |
| H57 | 19 | EF636783.1 | Highlands (Scotland) | modern | Western | Pérez-Espona et al. (2009) | 4 |  |
| H58 | 1 | EF636784.1 | Highlands (Scotland) | modern | Western | Pérez-Espona et al. (2009) | 4 |  |
| H59 | 9 | EF636785.1 | Highlands (Scotland) | modern | Western | Pérez-Espona et al. (2009) | 4 |  |
| H60 | 23 | EF636786.1 | Highlands (Scotland) | modern | Western | Pérez-Espona et al. (2009) | 4 |  |
| H61 | 9 | EF636787.1 | Highlands (Scotland) | modern | Western | Pérez-Espona et al. (2009) | 4 |  |
| H62 | 9 | EF636788.1 | Highlands (Scotland) | modern | Western | Pérez-Espona et al. (2009) | 4 |  |
| H63 | 18 | EF636789.1 | Highlands (Scotland) | modern | Western | Pérez-Espona et al. (2009) | 4 |  |
| H64 | 18 | EF636790.1 | Highlands (Scotland) | modern | Western | Pérez-Espona et al. (2009) | 4 |  |
| H65 | 1 | EF636791.1 | Highlands (Scotland) | modern | Western | Pérez-Espona et al. (2009) | 4 |  |
| H66 | 20 | EF636792.1 | Highlands (Scotland) | modern | Western | Pérez-Espona et al. (2009) | 4 |  |
| H67 | 58 | EF636793.1 | Highlands (Scotland) | modern | Western | Pérez-Espona et al. (2009) | 4 |  |
| H68 | 19 | EF636794.1 | Highlands (Scotland) | modern | Western | Pérez-Espona et al. (2009) | 4 |  |
| H69 | 58 | EF636795.1 | Highlands (Scotland) | modern | Western | Pérez-Espona et al. (2009) | 4 |  |
| H70 | 18 | EF636796.1 | Highlands (Scotland) | modern | Western | Pérez-Espona et al. (2009) | 4 |  |
| H71 | 19 | EF636797.1 | Highlands (Scotland) | modern | Western | Pérez-Espona et al. (2009) | 4 |  |
| H72 | 19 | EF636798.1 | Highlands (Scotland) | modern | Western | Pérez-Espona et al. (2009) | 4 |  |
| H73 | 58 | EF636799.1 | Highlands (Scotland) | modern | Western | Pérez-Espona et al. (2009) | 4 |  |
| H74 | 19 | EF636800.1 | Highlands (Scotland) | modern | Western | Pérez-Espona et al. (2009) | 4 |  |
| NO1 | 9 | JX861260 | Norway | ancient | Western | Rosvold et al. (2012) | 4 |  |
| NO2 | 9 | JX861261 | Norway | ancient | Western | Rosvold et al. (2012) | 4 |  |
| NO3 | 29 | JX861262 | Norway | ancient | Western | Rosvold et al. (2012) | 4 |  |
| NO4 | 18 | JX861263 | Norway | ancient | Western | Rosvold et al. (2012) | 4 |  |
| NO5 | 1 | JX861264 | Norway | ancient | Western | Rosvold et al. (2012) | 4 |  |
| NO6 | 59 | JX861265 | Norway | ancient | Western | Rosvold et al. (2012) | 4 |  |
| NO7 | 18 | JX861266 | Norway | ancient | Western | Rosvold et al. (2012) | 4 |  |
| NO8 | 79 | JX861267 | Norway | ancient | Western | Rosvold et al. (2012) | 4 |  |
| NO9 | 80 | JX861268 | Norway | ancient | Western | Rosvold et al. (2012) | 4 |  |
| NO10 | 81 | JX861269 | Norway | ancient | Western | Rosvold et al. (2012) | 4 |  |
| AA9 | 21 | DQ520200.1 | France | modern | Western | Skog et al. (2009) | 4 |  |
| AA9 | 21 | DQ520200.1 | France | modern | Western | Skog et al. (2009) | 4 |  |
| AD2 | 12 | DQ520201.1 | France | modern | Western | Skog et al. (2009) | 4 |  |
| AD2 | 12 | DQ520201.1 | France | modern | Western | Skog et al. (2009) | 4 |  |
| AD1 | 23 | DQ520202.1 | France | modern | Western | Skog et al. (2009) | 4 |  |
| AA3 | 16 | DQ520203.1 | France | modern | Western | Skog et al. (2009) | 4 |  |
| AA2 | 18 | DQ520204.1 | Spain | modern | Western | Skog et al. (2009) | 4 |  |
| AD7 | 24 | DQ520205.1 | Romania | modern | Western | Skog et al. (2009) | 4 |  |
| AB1 | 25 | DQ520206.1 | Spain | modern | Western | Skog et al. (2009) | 4 |  |
| AB1 | 25 | DQ520206.1 | Spain | modern | Western | Skog et al. (2009) | 4 |  |
| AA10 | 26 | DQ520207.1 | Spain | modern | Western | Skog et al. (2009) | 4 |  |
| AD9 | 1 | DQ520208.1 | UK | modern | Western | Skog et al. (2009) | 4 |  |
| AC5 | 27 | DQ520209.1 | Germany | modern | Western | Skog et al. (2009) | 4 |  |
| AC7 | 27 | DQ520210.1 | Germany | modern | Western | Skog et al. (2009) | 4 |  |
| AD8 | 19 | DQ520211.1 | UK | modern | Western | Skog et al. (2009) | 4 |  |
| AD6 | 18 | DQ520212.1 | UK | modern | Western | Skog et al. (2009) | 4 |  |
| AD5 | 9 | DQ520213.1 | UK | modern | Western | Skog et al. (2009) | 4 |  |
| AD5 | 9 | DQ520213.1 | UK | modern | Western | Skog et al. (2009) | 4 |  |
| AC6 | 27 | DQ520214.1 | Germany | modern | Western | Skog et al. (2009) | 4 |  |
| AA7 | 1 | DQ520215.1 | Norway | modern | Western | Skog et al. (2009) | 4 |  |
| AA7 | 1 | DQ520215.1 | Norway | modern | Western | Skog et al. (2009) | 4 |  |
| AD4 | 20 | DQ520216.1 | UK | modern | Western | Skog et al. (2009) | 4 |  |
|  | 20 | DQ520216.1 | UK | modern | Western | Skog et al. (2009) | 4 |  |
| AD10 | 1 | DQ520217.1 | UK | modern | Western | Skog et al. (2009) | 4 |  |
| AC2 | 28 | DQ520218.1 | Sweden | modern | Western | Skog et al. (2009) | 4 |  |
| AA1 | 18 | DQ520219.1 | Norway | modern | Western | Skog et al. (2009) | 4 |  |
| AA1 | 18 | DQ520219.1 | Spain | modern | Western | Skog et al. (2009) | 4 |  |
| AC3 | 9 | DQ520220.1 | Norway | modern | Western | Skog et al. (2009) | 4 |  |
| AC1 | 9 | DQ520221.1 | Norway | modern | Western | Skog et al. (2009) | 4 |  |
| AC1 | 9 | DQ520221.1 | Norway | modern | Western | Skog et al. (2009) | 4 |  |
| AA8 | 1 | DQ520222.1 | France | modern | Western | Skog et al. (2009) | 4 |  |
| AC4 | 29 | DQ520223.1 | Norway | modern | Western | Skog et al. (2009) | 4 |  |
| AC4 | 29 | DQ520223.1 | Norway | modern | Western | Skog et al. (2009) | 4 |  |
| AD3 | 30 | DQ520224.1 | Italy | modern | Western | Skog et al. (2009) | 4 |  |
| AB2 | 31 | DQ520225.1 | Italy | modern | Western | Skog et al. (2009) | 4 |  |
| AB3 | 31 | DQ520226.1 | Germany | modern | Western | Skog et al. (2009) | 4 |  |
| AA5 | 32 | DQ520227.1 | Spain | modern | Western | Skog et al. (2009) | 4 |  |
| AA6 | 33 | DQ520228.1 | Italy | modern | Western | Skog et al. (2009) | 4 |  |
| AA4 | 34 | DQ520229.1 | Spain | modern | Western | Skog et al. (2009) | 4 |  |
| AB4 | 35 | DQ520230.1 | Spain | modern | Western | Skog et al. (2009) | 4 |  |
| AB6 | 36 | DQ520231.1 | Spain | modern | Western | Skog et al. (2009) | 4 |  |
| AB6 | 36 | DQ520231.1 | Spain | modern | Western | Skog et al. (2009) | 4 |  |
| AB5 | 37 | DQ520232.1 | Spain | modern | Western | Skog et al. (2009) | 4 |  |
| AB7 | 38 | DQ520233.1 | Spain | modern | Western | Skog et al. (2009) | 4 |  |
| CB4 | 39 | DQ520234.1 | Italy | modern | Eastern | Skog et al. (2009) | 4 |  |
| CB5 | 39 | DQ520235.1 | Romania | modern | Eastern | Skog et al. (2009) | 4 |  |
| CB5 | 39 | DQ520235.1 | Romania | modern | Eastern | Skog et al. (2009) | 4 |  |
| CB5 | 39 | DQ520235.1 | CzechRepublic | modern | Eastern | Skog et al. (2009) | 4 |  |
| CB5 | 39 | DQ520235.1 | CzechRepublic | modern | Eastern | Skog et al. (2009) | 4 |  |
| CB5 | 39 | DQ520235.1 | CzechRepublic | modern | Eastern | Skog et al. (2009) | 4 |  |
| CB5 | 39 | DQ520235.1 | Italy | modern | Eastern | Skog et al. (2009) | 4 |  |
| CB5 | 39 | DQ520235.1 | Italy | modern | Eastern | Skog et al. (2009) | 4 |  |
| CB3 | 39 | DQ520236 | Italy | modern | Eastern | Skog et al. (2009) | 4 |  |
| CB2 | 39 | DQ520237.1 | Italy | modern | Eastern | Skog et al. (2009) | 4 |  |
| CD1 | 40 | DQ520238.1 | Romania | modern | Eastern | Skog et al. (2009) | 4 |  |
| CD1 | 40 | DQ520238.1 | Romania | modern | Eastern | Skog et al. (2009) | 4 |  |
| CD1 | 40 | DQ520238.1 | Hungary | modern | Eastern | Skog et al. (2009) | 4 |  |
| CD1 | 40 | DQ520238.1 | CzechRepublic | modern | Eastern | Skog et al. (2009) | 4 |  |
| CD1 | 40 | DQ520238.1 | Italy | modern | Eastern | Skog et al. (2009) | 4 |  |
| CD1 | 40 | DQ520238.1 | Italy | modern | Eastern | Skog et al. (2009) | 4 |  |
| CD6 | 41 | DQ520239.1 | Romania | modern | Eastern | Skog et al. (2009) | 4 |  |
| CD4 | 42 | DQ520240.1 | Bulgaria | modern | Eastern | Skog et al. (2009) | 4 |  |
| CD4 | 42 | DQ520240.1 | Romania | modern | Eastern | Skog et al. (2009) | 4 |  |
| CD2 | 40 | DQ520241.1 | Romania | modern | Eastern | Skog et al. (2009) | 4 |  |
| CD3 | 43 | DQ520242.1 | Hungary | modern | Eastern | Skog et al. (2009) | 4 |  |
| CD5 | 44 | DQ520243.1 | Bulgaria | modern | Eastern | Skog et al. (2009) | 4 |  |
| CB1 | 40 | DQ520244.1 | Italy | modern | Eastern | Skog et al. (2009) | 4 |  |
| CB1 | 40 | DQ520244.1 | Italy | modern | Eastern | Skog et al. (2009) | 4 |  |
| CA4 | 45 | DQ520245.1 | Romania | modern | Eastern | Skog et al. (2009) | 4 |  |
| CA4 | 45 | DQ520245.1 | Italy | modern | Eastern | Skog et al. (2009) | 4 |  |
| CA3 | 46 | DQ520246.1 | Romania | modern | Eastern | Skog et al. (2009) | 4 |  |
| CA3 | 46 | DQ520246.1 | CzechRepublic | modern | Eastern | Skog et al. (2009) | 4 |  |
| CA3 | 46 | DQ520246.1 | CzechRepublic | modern | Eastern | Skog et al. (2009) | 4 |  |
| CA5 | 47 | DQ520247.1 | Serbia | modern | Eastern | Skog et al. (2009) | 4 |  |
| CA5 | 47 | DQ520247.1 | Hungary | modern | Eastern | Skog et al. (2009) | 4 |  |
| CA1 | 47 | DQ520248.1 | Bulgaria | modern | Eastern | Skog et al. (2009) | 4 |  |
| CA1 | 47 | DQ520248.1 | Romania | modern | Eastern | Skog et al. (2009) | 4 |  |
| CA1 | 47 | DQ520248.1 | Serbia | modern | Eastern | Skog et al. (2009) | 4 |  |
| CA1 | 47 | DQ520248.1 | Italy | modern | Eastern | Skog et al. (2009) | 4 |  |
| CA2 | 47 | DQ520249.1 | Italy | modern | Eastern | Skog et al. (2009) | 4 |  |
| CA6 | 48 | DQ520250.1 | Romania | modern | Eastern | Skog et al. (2009) | 4 |  |
| BB | 49 | DQ520251.1 | Italy | modern | Sardinia | Skog et al. (2009) | 4 |  |
| BA | 49 | DQ520252.1 | Italy | modern | Sardinia | Skog et al. (2009) | 4 |  |
| BE | 49 | DQ520253.1 | Italy | modern | Sardinia | Skog et al. (2009) | 4 |  |
| BF | 49 | DQ520254.1 | Italy | modern | Sardinia | Skog et al. (2009) | 4 |  |
| BD | 11 | DQ520255.1 | Italy | modern | Sardinia | Skog et al. (2009) | 4 |  |
| BC | 11 | DQ520256.1 | Italy | modern | Sardinia | Skog et al. (2009) | 4 |  |
| BC | 11 | DQ520256.1 | Spain | modern | Sardinia | Skog et al. (2009) | 4 |  |
| APL10 | 138 | ku877699 | Applecross (Scotland) | LATE-IRON | Western | Stanton et al. (2016) | 4 |  |
| B09 | 139 | ku877700 | Orkney (Scotland) | NEOLITHIC | Western | Stanton et al. (2016) | 4 |  |
| B11 | 139 | ku877700 | Orkney (Scotland) | NEOLITHIC | Western | Stanton et al. (2016) | 4 |  |
| NT29 | 139 | ku877700 | Outer (Scotland) | NEOLITHIC | Western | Stanton et al. (2016) | 4 |  |
| NT55 | 139 | ku877700 | Outer (Scotland) | IRON | Western | Stanton et al. (2016) | 4 |  |
| CN01 | 140 | ku877701 | Inner (Scotland) | MESOLITHIC | Western | Stanton et al. (2016) | 4 |  |
| CN03 | 140 | ku877701 | Inner (Scotland) | MESOLITHIC | Western | Stanton et al. (2016) | 4 |  |
| CN04 | 140 | ku877701 | Inner (Scotland) | MESOLITHIC | Western | Stanton et al. (2016) | 4 |  |
| CN05 | 140 | ku877701 | Inner (Scotland) | MESOLITHIC | Western | Stanton et al. (2016) | 4 |  |
| CN06 | 140 | ku877701 | Inner (Scotland) | MESOLITHIC | Western | Stanton et al. (2016) | 4 |  |
| CS01 | 140 | ku877701 | Inner (Scotland) | MESOLITHIC | Western | Stanton et al. (2016) | 4 |  |
| CS02 | 140 | ku877701 | Inner (Scotland) | MESOLITHIC | Western | Stanton et al. (2016) | 4 |  |
| CS04 | 140 | ku877701 | Inner (Scotland) | MESOLITHIC | Western | Stanton et al. (2016) | 4 |  |
| PRO1 | 140 | ku877701 | Inner (Scotland) | MESOLITHIC | Western | Stanton et al. (2016) | 4 |  |
| RS02 | 140 | ku877701 | Inner (Scotland) | MESOLITHIC | Western | Stanton et al. (2016) | 4 |  |
| RS03 | 140 | ku877701 | Inner (Scotland) | MESOLITHIC | Western | Stanton et al. (2016) | 4 |  |
| DS02 | 141 | ku877702 | Outer (Scotland) | IRON | Western | Stanton et al. (2016) | 4 |  |
| DS05 | 141 | ku877702 | Outer (Scotland) | IRON | Western | Stanton et al. (2016) | 4 |  |
| DS10 | 141 | ku877702 | Outer (Scotland) | NORSE | Western | Stanton et al. (2016) | 4 |  |
| MH10 | 141 | ku877702 | Orkney (Scotland) | IRON | Western | Stanton et al. (2016) | 4 |  |
| MH14 | 141 | ku877702 | Orkney (Scotland) | IRON | Western | Stanton et al. (2016) | 4 |  |
| MH16 | 141 | ku877702 | Orkney (Scotland) | IRON | Western | Stanton et al. (2016) | 4 |  |
| MH28 | 141 | ku877702 | Orkney (Scotland) | IRON | Western | Stanton et al. (2016) | 4 |  |
| MH32 | 141 | ku877702 | Orkney (Scotland) | IRON | Western | Stanton et al. (2016) | 4 |  |
| MH33 | 141 | ku877702 | Orkney (Scotland) | IRON | Western | Stanton et al. (2016) | 4 |  |
| MH35 | 141 | ku877702 | Orkney (Scotland) | IRON | Western | Stanton et al. (2016) | 4 |  |
| NT3 | 141 | ku877702 | Outer (Scotland) | BEAKERVII | Western | Stanton et al. (2016) | 4 |  |
| NT32 | 141 | ku877702 | Outer (Scotland) | NEOLITHIC | Western | Stanton et al. (2016) | 4 |  |
| NT34 | 141 | ku877702 | Outer (Scotland) | NEOLITHIC | Western | Stanton et al. (2016) | 4 |  |
| NT35 | 141 | ku877702 | Outer (Scotland) | NEOLITHIC | Western | Stanton et al. (2016) | 4 |  |
| NT36 | 141 | ku877702 | Outer (Scotland) | NEOLITHIC | Western | Stanton et al. (2016) | 4 |  |
| NT5 | 141 | ku877702 | Outer (Scotland) | BEAKERVII | Western | Stanton et al. (2016) | 4 |  |
| NT53 | 141 | ku877702 | Outer (Scotland) | IRON | Western | Stanton et al. (2016) | 4 |  |
| NT57 | 141 | ku877702 | Orkney (Scotland) | IRON | Western | Stanton et al. (2016) | 4 |  |
| NT8 | 141 | ku877702 | Outer (Scotland) | BEAKERVII | Western | Stanton et al. (2016) | 4 |  |
| NT9 | 141 | ku877702 | Outer (Scotland) | BEAKERVII | Western | Stanton et al. (2016) | 4 |  |
| LON44 | 78 | ku877703 | Orkney (Scotland) | NEOLITHIC | Western | Stanton et al. (2016) | 4 |  |
| MOA97 | 138 | ku877704 | Orkney (Scotland) | BRONZE | Western | Stanton et al. (2016) | 4 |  |
| NT1 | 142 | ku877705 | Outer (Scotland) | BEAKERVII | Western | Stanton et al. (2016) | 4 |  |
| NT4 | 143 | ku877706 | Outer (Scotland) | BEAKER | Western | Stanton et al. (2016) | 4 |  |
| RS01 | 78 | ku877707 | Inner (Scotland) | MESOLITHIC | Western | Stanton et al. (2016) | 4 |  |
| NT52 | 144 | ku877708 | Outer (Scotland) | LATE-IRON | Western | Stanton et al. (2016) | 4 |  |
| DS03 | 145 | ku877709 | Outer (Scotland) | EARLY-NORSE | Western | Stanton et al. (2016) | 4 |  |
| DS07 | 146 | ku877710 | Outer (Scotland) | EARLY-NORSE | Western | Stanton et al. (2016) | 4 |  |
| DS09 | 147 | ku877711 | Outer (Scotland) | EARLY-NORSE | Western | Stanton et al. (2016) | 4 |  |
| DS08 | 78 | ku877712 | Outer (Scotland) | LATE-NORSE | Western | Stanton et al. (2016) | 4 |  |
| CGG-3-014525 | 2 | MF872239 | Galicia (Spain) | 38000 | Eastern | This study | 1,2,3,4 | 42.73,-7.027 |
| CGG-3-014530 | 2 | MF872238 | Galicia (Spain) | 38000 | Eastern | This study | 1,2,3,4 | 42.73,-7.027 |
| CGG-3-014535 | 3 | MF872242 | Galicia (Spain) | 38000 | Eastern | This study | 1,2,3,4 | 42.73,-7.027 |
| CGG-3-014537 | 4 | MF872243 | Galicia (Spain) | 38000 | Eastern | This study | 1,2,3,4 | 42.73,-7.027 |
| CGG_3_014526 | 5 | MF872244 | Galicia (Spain) | 38000 | Western | This study | 1,2,3,4 | 42.73,-7.027 |
| CGG_3_014533 | 6 | MF872246 | Galicia (Spain) | 38000 | Western | This study | 1,2,3,4 | 42.73,-7.027 |
| CGG_3_014536 | 7 | MF872241 | Galicia (Spain) | 38000 | Eastern | This study | 1,2,3,4 | 42.73,-7.027 |
| CGG_3_014538 | 8 | MF872249 | Galicia (Spain) | 38000 | Western | This study | 1,2,3,4 | 42.73,-7.027 |
| CGG_3_016150 | 9 | MF872240 | Galicia (Spain) | 38000 | Western | This study | 1,2,3,4 | 42.73,-7.027 |
| CGG_3_016151 | 10 | MF872245 | Galicia (Spain) | 38000 | Western | This study | 1,2,3,4 | 42.73,-7.027 |
| ZMUC_2 | 59 | MF872247 | Denmark | Holocene | Western | This study | 1,2,3,4 | 56.088,12.455 |
| ZMUC_3 | 153 | MF872236/ MF872237 | Denmark | Holocene | Western | This study | 1,2,3,4 | 55.146,15.033 |
| ZMUC_5 | 9 | MF872248 | Denmark | Holocene | Western | This study | 1,2,3,4 | 55.111,11.874 |
